# Adaptive control paradigm for photovoltaic and solid oxide fuel cell in a grid-integrated hybrid renewable energy system

**DOI:** 10.1371/journal.pone.0173966

**Published:** 2017-03-22

**Authors:** Sidra Mumtaz, Laiq Khan

**Affiliations:** Department of Electrical Engineering, COMSATS Institute of Information Technology, Abbottabad, Khyber Pakhtunkhwa, Pakistan; Chongqing University, CHINA

## Abstract

The hybrid power system (HPS) is an emerging power generation scheme due to the plentiful availability of renewable energy sources. Renewable energy sources are characterized as highly intermittent in nature due to meteorological conditions, while the domestic load also behaves in a quite uncertain manner. In this scenario, to maintain the balance between generation and load, the development of an intelligent and adaptive control algorithm has preoccupied power engineers and researchers. This paper proposes a Hermite wavelet embedded NeuroFuzzy indirect adaptive MPPT (maximum power point tracking) control of photovoltaic (PV) systems to extract maximum power and a Hermite wavelet incorporated NeuroFuzzy indirect adaptive control of Solid Oxide Fuel Cells (SOFC) to obtain a swift response in a grid-connected hybrid power system. A comprehensive simulation testbed for a grid-connected hybrid power system (wind turbine, PV cells, SOFC, electrolyzer, battery storage system, supercapacitor (SC), micro-turbine (MT) and domestic load) is developed in Matlab/Simulink. The robustness and superiority of the proposed indirect adaptive control paradigm are evaluated through simulation results in a grid-connected hybrid power system testbed by comparison with a conventional PI (proportional and integral) control system. The simulation results verify the effectiveness of the proposed control paradigm.

## 1. Introduction

The global electricity demand is expected to increase 49% from 2007 to 2035 [1]. At present, most of the electricity demand is met by fossil fuels. These fossil fuels have caused adverse environmental effects, and their reserves are declining with the passage of time. Moreover, the rapid increase in electricity demand and scarcity of fossil fuels increase the cost of electricity. Thus, it is essential to endeavor to decrease greenhouse gas emissions and obtain affordable long-term sustainable energy sources. Recently, renewable energy has gained much more attention as an alternative energy. Renewable energy is clean, is sustainable, is economical and never runs out. The power from renewable energy is at the mercy of meteorological conditions. Thus, any standalone renewable energy source is unable to supply reliable and sustainable power. Therefore, multiple renewable and non-renewable power sources are incorporated to form a hybrid power system. Currently, in the energy market, hybrid power systems based on renewable energy have paved an attractive approach to produce electricity [2]. A PV power based stochastic optimization framework is used to manage the energy of a smart home with plug-in electric vehicle (PEV) storage [3]. Moreover, to achieve the accuracy, PV power and home load demand are also forecasted using a radial basis function neural network (RBF-NN). A wind/PV/fuel cell generation-based HPS is presented for a typical home in the US Pacific Northwest [4]. A standalone application of HPS contains wind, PV cells, fuel cells (FC), an electrolyzer and a battery, which are integrated through an AC link bus [5]. To obtain the maximum advantage from renewable energy, wind and PV cells are considered as primary sources of the HPS. The FC/electrolyzer and battery are used as a backup system. However, the stated HPS operates as a standalone system. A PV, FC and ultra-capacitor (UC)-based standalone HPS is used to supply sustained power [6]. During adequate irradiance, the excess power generated by the PV system is fed to the electrolyzer. Conversely, when the PV system is unable to meet the load, the FC tries to satisfy the load, but if load power deficiency still exists, then UC supplies the auxiliary power. However, the standalone application makes the HPS operate off-grid—i.e., not connected to any distribution grid. Thus, standalone application limits the scope of the stated HPS. A PV/electrolyzer coupled with a SOFC based on energy and exergy is developed to supply the electricity to a residential load, but the maximum obtained efficiencies for energy and exergy are 55.7% and 49%, respectively [7]. In the mutable environment, to increase the output efficiency of the PV system, a maximum power point tracking (MPPT) algorithm is required to search the optimal operating voltage and/or current of the PV system. The nonlinear behavior of the current—voltage curve of the PV system makes MPPT a more challenging issue. In the literature, various techniques have been proposed depending upon complexity, convergence speed, control, stability and cost. The most commonly used techniques are perturb and observe (P&O), incremental conductance (IC), constant voltage (CV) and constant current (CC) algorithms [8], [9], [10], [11]. Among them, P&O is widely adopted due to its simplicity and easy hardware implementation, but once the maximum power point is achieved, the system keeps oscillating around this power point.

SOFC is an alternative versatile energy source, because it converts chemical energy into electrical energy with negligible emissions. However, SOFC presents a challenging control issue during load following due to its sluggish dynamics, nonlinearity and strict operating constraints. A sudden change in load power causes hydrogen starvation in the SOFC, i.e., the partial pressure of oxygen drops significantly, which lowers the cell voltage rapidly and hence shortens the life of the SOFC. Moreover, this fuel starvation also permanently damages the SOFC. Thus, an efficient control system is needed to ensure that the SOFC satisfies the dynamic load with high operating efficiency. Two types of control strategies exist for SOFC. One is to control the input hydrogen in proportion to the stack current, and the other is to maintain a constant voltage at the SOFC terminals [12]. In the literature, several control strategies are used for SOFC, which include a multi-loop feedback control, a master control, PID feedback control and model predictive control, but these techniques are computationally slow, complex and difficult to implement [13], [14], [15], [16], [17].

Similarly, numerous intelligent and modern soft computing techniques are widely used to obtain the PV MPPT and swift response of SOFC, which include evolutionary algorithms, artificial neural network (ANN), fuzzy logic and their hybrids. Evolutionary algorithms (DE, GA, PSO, etc.) are stochastic processes that are quite efficient to optimize real-valued, nonlinear and multi-objective problems [18], [19], [20]. However, evolutionary algorithms need more research and technological development for the appropriate selection of control parameters, initial values, solution archive and locality of the search space. ANN is a non-parametric and nonlinear regression soft computing technique. The learning capability of ANN provides “implicit knowledge” in the form of hidden neurons [21]. However, in ANN, the selection of optimal initial values of the weights, centers and spreads of the hidden unit is a crucial issue. The fuzzy logic control system is robust and somewhat simple to design, because no precise mathematical model is needed. However, the appropriate selection of the fuzzy inference system is quite important for accurate performance of the fuzzy logic control system [22]. However, the synergy of two paradigms—i.e., ANN and fuzzy logic—offers another prevailing artificial intelligence technique called NeuroFuzzy. This control scheme has good generalization capability, has low complexity and is easy to implement [23]. NeuroFuzzy amalgamates the explicit knowledge of fuzzy logic with the implicit knowledge of ANN [24]. However, the inherent drawback of the NeuroFuzzy system is that it not only becomes trapped in local minima of the search space but also has long computational time. Moreover, due to the linear consequent part, the classical NeuroFuzzy network becomes inefficient to handle system nonlinearities. These inherent issues are mitigated by introducing wavelets into the NeuroFuzzy network. Wavelet inclusion significantly improves the computational speed of the NeuroFuzzy network. The wavelet-based network is considered as an optimal approximator, because it explores a small number of data chunks to achieve precision [25], [26].

However, when a nonlinear system such as the PV system has unknown and uncertain parameters due to the fluctuating environment, the abovementioned control systems are no longer applicable. The adaptive control paradigm is suitable to sustain the reliable performance of a nonlinear system in real time even in the occurrence of unknown and uncertain variation [27]. There are two different classes of adaptive control: direct and indirect [28]. A direct adaptive control system directly updates the controller parameters without involving the explicit identification of the unknown plant. However, direct adaptive control fails to capture the instantaneous dynamics of the nonlinear system [29], [30], [31]. The indirect adaptive control (or self-tuning regulator) is more appropriate to work with a fluctuating environment, because it adaptively identifies the model of the plant used to calculate the controller parameters [32], [33], [34].

To address all the afore mentioned hitches, an efficient Hermite wavelet embedded NeuroFuzzy indirect adaptive MPPT control scheme for PV systems and an effective Hermite wavelet incorporated NeuroFuzzy indirect adaptive control scheme for SOFC systems integrated with HPS is proposed. In the stated HPS, tracking the maximum power point (MPP) for the PV system and obtaining the swift response of the SOFC are quite perplexing issues, because this system is greatly characterized by nonlinearity. The nonlinearity arises due to the erratic load, dynamic solar radiation and inconsistent temperature.

Given the above interpretation, it is observed that the proposed adaptive control paradigm possesses the following characteristics:

- The Hermite wavelet embedded NeuroFuzzy indirect adaptive MPPT control of PV is characterized by nonlinearity, which operates on the instantaneously captured nonlinear dynamics of the system.
- The Hermite wavelet incorporated NeuroFuzzy indirect adaptive control of SOFC is embodied to respond swiftly to the nonlinear identified dynamics of the system due of sudden load changes.
- The Hermite wavelet has strong identification capability, which is exploited by the adaptive controllers for PV maximum power point tracking and SOFC output power control.
- The NeuroFuzzy algorithm is characterized by “explicit knowledge” by virtue of fuzzy logic and “implicit knowledge” by virtue of the neural network, which makes the adaptive control paradigm transparent and evolvable.

The rest of the paper is organized in six main sections. Section 2 presents the mathematical modeling of the proposed hybrid power system. Section 3 gives the detailed mathematical description of the proposed control strategies. Simulation results are discussed in section 4. Section 5 concludes the outcomes of this research work.

## 2. System overview and model description

**Fig 1:**
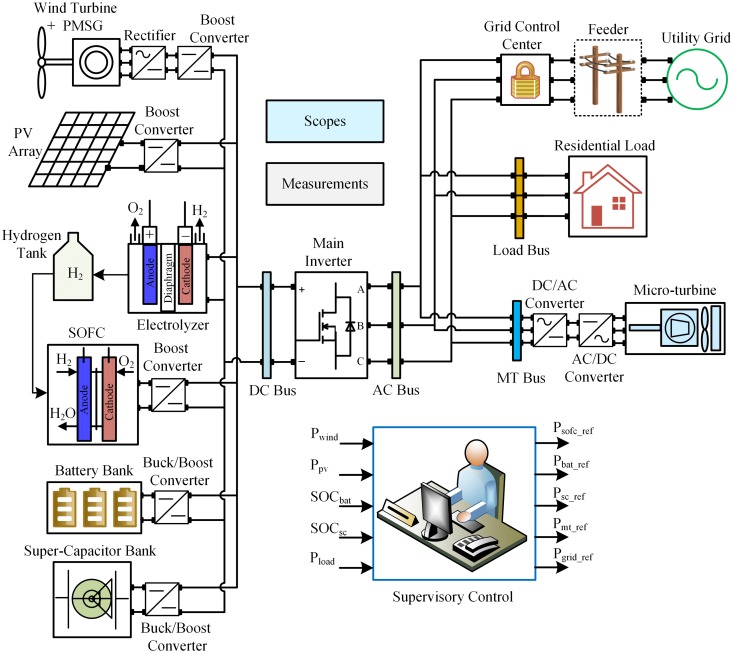
Hybrid power system.

The configuration of the stated HPS is shown in Fig 1, which consists of wind turbine, PV cells, SOFC, electrolyzer, battery, SC, MT and residential load connected to the utility grid. Two buses are connected through the main inverter in the suggested HPS—i.e., DC bus and AC bus. HPS contains multiple power generation sources to compensate for the components’ respective strengths and weaknesses. A permanent magnet synchronous generator (PMSG)-based wind turbine is connected to the DC bus via a rectifier. The PV system is connected to the DC bus via a boost converter, which boosts the PV output voltage to the DC bus voltage. The electrolyzer utilizes surplus power to produce the hydrogen, which is used by the SOFC, so the electrolyzer acts as an energy buffer. The battery is used as a backup in the HPS and is connected to the DC bus via a bidirectional buck/boost converter. The battery is quite efficient when low and steady power levels are needed. The SC is also used as a backup source and is connected to the DC bus via a bidirectional buck/boost converter. SC can process several hundred thousand charge/discharge cycles compared to only a few thousand charge/discharge cycles for the battery. The MT is operated as a standby source in HPS, which is connected to the AC bus through back-to-back AC/DC and then DC/AC converters.

### 2.1 Mathematical modeling of hybrid power system components

#### 2.1.1 Wind generation system

**Fig 2:**
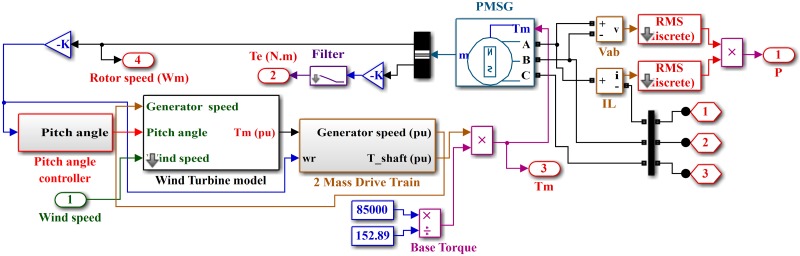
Wind generation system with PMSG.

**Fig 3:**
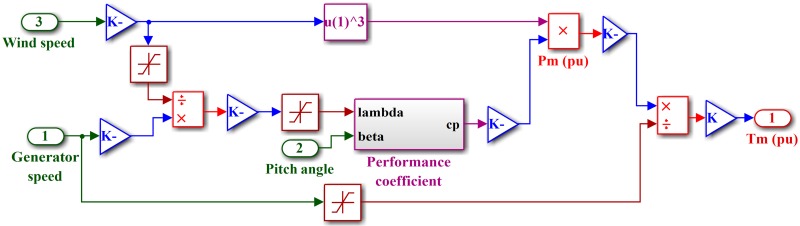
Simulink model wind turbine.

The PMSG-based wind generation system is shown in Fig 2. The wind turbine model is shown in Fig 3. The wind turbine produces mechanical energy, which is used to run a PMSG to obtain the electrical energy [35]. The output power captured by the wind turbine is given as

$$P_{m}=c_{p}(\lambda ,\beta )\frac{\rho A}{2}v^{3}$$ (1)

where *P*_*m*_ is the mechanical output power, *c*_*p*_ is the performance coefficient, *ρ* is the air density (kg/m^3^), *A* is the swept area of blades, *v* is the wind speed, *λ* is the tip speed ratio (TSR), and *β* is the blade pitch angle. TSR is the ratio between the linear speed of the blade tips and the rotational speed of the turbine and can be calculated as

$$\lambda =\frac{\omega_{m}R}{v}$$ (2)

where *R* is rotor radius, and *ω*_*m*_ is the mechanical speed of the generator. The performance coefficient is the nonlinear function of the TSR and blade pitch angle, which determines the power captured efficiency of the turbine and can be measured as follows:

$$c_{p}(\lambda ,\beta )={\text{c}}_{1}({\text{c}}_{2}/\lambda_{i}-{\text{c}}_{3}\beta -{\text{c}}_{4}){\text{e}}^{-{\text{c}}_{5}/\lambda_{i}}+c_{6}\lambda$$ (3)

with

$$\frac{1}{\lambda_{i}}=\frac{1}{\lambda +0.08\beta }-\frac{0.035}{\beta^{3}+1}$$ (4)

where the coefficients are c_1_ = 0.5176, c_2_ = 116, c_3_ = 0.4, c_4_ = 5, c_5_ = 21 and c_6_ = 0.0068. The maximum value of *c*_*p*_(*λ*, *β*) is obtained at *β* = 0° and *λ* = 8.1. The electrical torque produced by the PMSG rotor is calculated as

$$T_{e}=\left(\frac{3}{2}\right)\left(\frac{p}{2}\right)\left[\left(L_{d}-L_{q}\right)i_{q}i_{d}-\lambda_{m}i_{q}\right]$$ (5)

where *T*_*e*_ is the electrical torque, *p* is the number of poles, *L*_*d*_ is the inductance of the d-axis, *L*_*q*_ is the inductance of the q-axis, *i*_*d*_ is the current produced by the d-axis, *i*_*q*_ is the current produced by the q-axis, and *λ*_*m*_ is the amplitude of flux linkages. The important parameters of the wind turbine are listed in Table 1.

**Table 1:** Parameters of wind turbine.

Wind turbine	
**Type**	nED-100
**Base wind speed**	10 m/s
**Rotor speed**	33–67 rpm
**Drive train**	2-mass model
**Pitch angle**	0°
**Rated power**	100 kW

#### 2.1.2 Photovoltaic array

**Fig 4:**
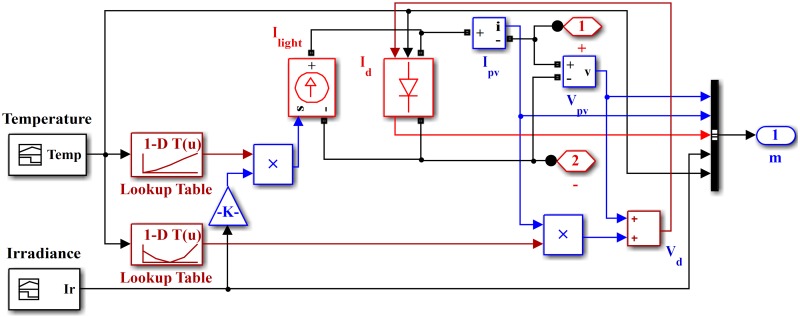
Simulink model of PV array.

The PV cell voltage and current vary with changing solar radiation and atmospheric temperature. The PV array current can be calculated as follows:

$$I_{pv}=I_{light}-I_{d}-\frac{V_{d}}{R_{shunt}}$$ (6)

where *I*_*pv*_ is the PV array generated current, *I*_*light*_ is the incident light current, *I*_*d*_ is the diode current, *V*_*d*_ is the diode voltage, and *R*_*shunt*_ is the shunt resistance, which represents the leakage current. The voltage of the PV array is calculated as

$$V_{pv}=n_{s}\left(\frac{\alpha \;\tau \;T}{q}\right)\text{ln}\left\{\frac{I_{s}-I_{pv}+n_{p}}{n_{p}\tilde{I}}\right\}-\frac{n_{s}}{n_{p}}I_{pv}R$$ (7)

where *V*_*pv*_ is the PV array voltage, *n*_*s*_ is the number of series-connected cells, *n*_*p*_ is the number of parallel connected cells, *T* is the cell temperature, *α* is the temperature coefficient, *τ* is Boltzmann’s constant, *q* is the charge of an electron, *I*_*s*_ is the short-circuit current, $\tilde{I}$ is the diode saturation current, and *R* is a series-connected resistance. The Simulink model of the PV array is shown in Fig 4. All parameters of the PV array are given in Table 2.

**Table 2:** Parameters of PV array.

PV Array	
**Type**	SunPower SPR-305-WHT
**Module unit**	305 W @ 1 kW/m^2^, 25°C
**`Number of series string**	13
**Number of parallel string**	66
**Power rating**	305 × 13 × 66 ≈ 262 kW

#### 2.1.3 Solid oxide fuel cell

**Fig 5:**
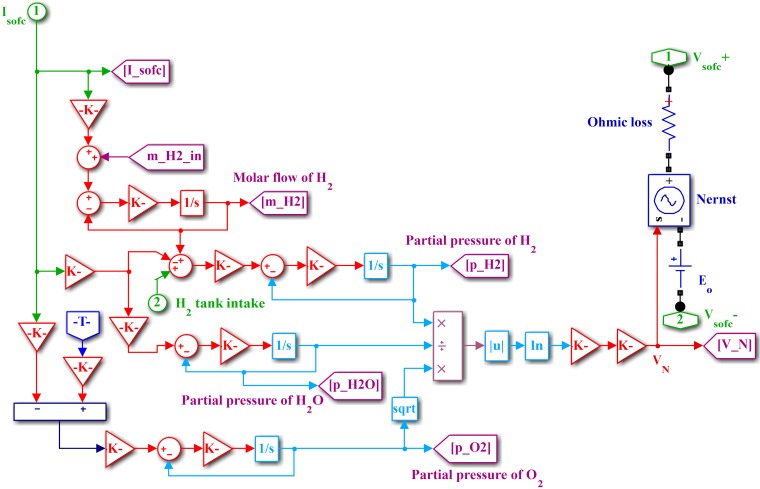
Simulink model of SOFC.

SOFC is an electrochemical conversion source that directly generates electricity by oxidizing the fuel. The fuel most commonly used by SOFC is hydrogen. The molar flow of hydrogen is calculated as

$$m_{H_{2}}^{ref}=\left(\frac{n_{s}I_{sofc}}{2F}\right)=2kI_{sofc}$$ (8)

where $m_{H_{2}}^{ref}$ is the molar flow of hydrogen, *n*_*s*_ is the number of series cells, *I*_*sofc*_ is the SOFC current, *F* is Faraday’s constant, and *k* is constant. The output voltage of SOFC is given as

$$V_{sofc}=V_{N}-V_{A}-V_{O}-V_{C}$$ (9)

where *V*_*sofc*_ is the SOFC output voltage, *V*_*N*_ is the Nernst potential, *V*_*A*_ is the activation polarization, *V*_*o*_ is the ohmic polarization, and *V*_*C*_ is the concentration polarization. The Nernst potential is given as

$$V_{N}=E_{0}+\frac{gT}{2F}\text{ln}\left\{\frac{\rho_{H_{2}}-\sqrt{\rho_{O_{2}}}}{\rho_{H_{2}O}}\right\}$$ (10)

where *g* is the gas constant, *T* is the cell temperature, $\rho_{H_{2}}$ is the partial pressure of hydrogen, $\rho_{O_{2}}$ is the partial pressure of oxygen, $\rho_{H_{2}O}$ is the partial pressure of water, and *E*_0_ is the reversible voltage. The Simulink model of SOFC is shown in Fig 5. Important SOFC parameters are listed in Table 3.

**Table 3:** Parameters of SOFC.

SOFC Array	
**Type**	Bloom Energy USA ES-5700
**Number of series cells**	768
**SOFC stack**	4 kW
**SOFC array**	5 × 10 = 50
**SOFC array power**	50 × 4 kW = 200 kW

#### 2.1.4 Electrolyzer

**Fig 6:**
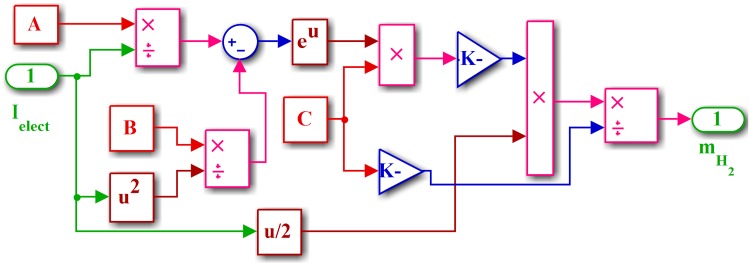
Simulink model of electrolyzer.

The electrolyzer dissociates water by using electrical energy to produce hydrogen and oxygen. The electrolyzer stack has several series-connected cells. The electrical efficiency of the electrolyzer is calculated as

$$\eta_{elect}=\eta_{I_{elect}}\times \eta_{V_{elect}}$$ (11)

where *η*_*elect*_ is the efficiency of the electrolyzer, $\eta_{I_{elect}}$ is the current efficiency, and $\eta_{V_{elect}}$ is the voltage efficiency. The amount of hydrogen produced (in mol) by the electrolyzer is given as

$$m_{H_{2}}=\frac{n_{s}I_{elect}}{2F}\eta_{elect}c$$ (12)

where $m_{H_{2}}$ is the amount of hydrogen produced, *n*_*s*_ is the number of series cells, *F* is Faraday’s constant, *I*_*elect*_ is the electrolyzer current, and *c* is a constant. The Simulink model of the electrolyzer is shown in Fig 6. The parameters of the electrolyzer are listed in Table 4.

**Table 4:** Parameters of electrolyzer.

Electrolyzer	
**Type**	QualeanQL-85000
**Rated power**	150 kW
**Rated voltage**	380 V
**Number of cells in stack**	30
**Number of electrolyzers**	6

#### 2.1.5 Battery

Lithium-ion batteries have high energy density; therefore, they are widely used to store energy in many industrial fields [36]. Battery voltage and state-of-charge (SOC) are the two most important parameters of a battery. Battery voltage is given as

$$V_{bat}=V_{oc}-R_{i}I_{bat}$$ (13)

where *V*_*bat*_ is the battery terminal voltage, *V*_*oc*_ is the open-circuit voltage, *R*_*i*_ is the internal resistance, and *I*_*bat*_ is the battery output current, which can be calculated as

$$I_{bat}=\frac{V_{oc}-\sqrt{V_{oc}^{2}-4R_{i}P}}{2R_{i}}$$ (14)

**Fig 7:**
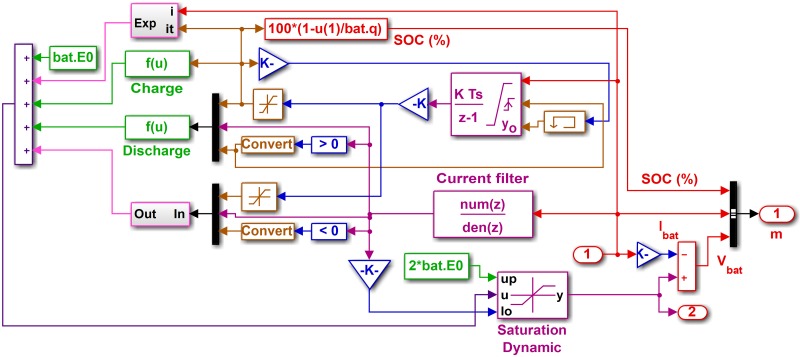
Simulink model of battery.

The SOC of a battery is calculated by the Coulomb counting method [35]:

$$SOC_{bat}=SOC_{bat}^{ini}-\int \frac{\eta I_{bat}}{q}dt$$ (15)

where *SOC*_*bat*_ is the SOC of the battery, $SOC_{bat}^{ini}$ is the initial SOC of the battery, *η* represents the charge or discharge mode, and *q* is the battery capacity (ampere hour). The estimation of SOC, capacity and internal resistance ensures the safe, reliable and efficient operation of lithium-ion batteries [36]. The Simulink model of the battery is shown in Fig 7. The battery parameters are given in Table 5.

**Table 5:** Parameters of battery.

Battery	
**Type**	CINCO FM/BB12100T
**Capacity**	200 Ah
**Voltage/string**	12 V
**Number of parallel strings**	3
**Number of series strings**	34
**Rated voltage**	12 × 34 ≈ 400 V

#### 2.1.6 Supercapacitor

**Fig 8:**
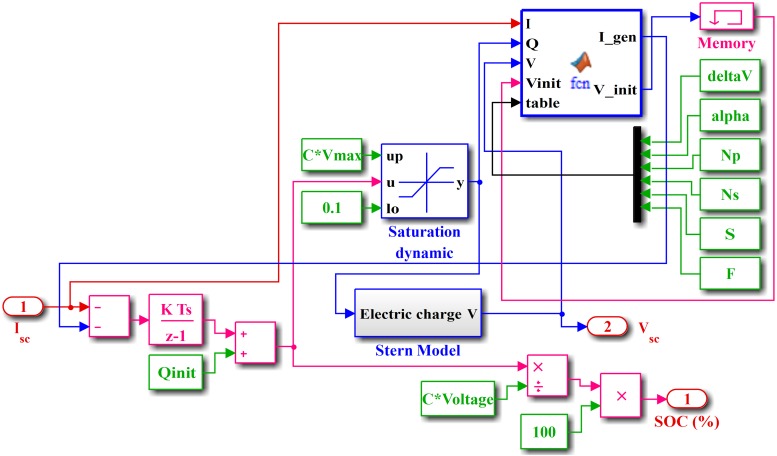
Simulink model of SC.

Supercapacitors are short-term energy storage devices with excellent power density and energy efficiency [37]. The SC has two important parameters, capacitance and resistance, which are assumed to be constant during charge/discharge cycles. The energy stored in the SC has a linear relationship with the square of the capacitor voltage as follows:

$$E_{sc}=0.5CV_{sc}^{2}=0.5C\left(\frac{P_{sc}}{I_{sc}}\right)$$ (16)

where *E*_*sc*_ is the energy stored in the SC, *C* is the capacitance, *V*_*sc*_ is the voltage of the SC, *P*_*sc*_ is the power of the SC, and *I*_*sc*_ is the current of the SC. The SOC of the SC can be calculated as follows:

$$SOC_{sc}=\frac{E_{sc}}{E_{sc}^{\text{max}}}$$ (17)

where *SOC*_*sc*_ is the SOC of the SC, and $E_{sc}^{\text{max}}$ is the maximum energy of the SC. The SOC and residual capacity of the SC can be estimated for reliable, resilient and safe operation [38], [39]. The voltage of the SC is given as

$$V_{sc}=R_{s}I_{sc}+\frac{1}{C}\int \left(I_{sc}-\frac{E_{sc}}{R_{p}}\right)\;dt+V_{sc}^{ini}$$ (18)

where *R*_*s*_ and *R*_*p*_ are equivalent series and parallel resistances, respectively, and $V_{sc}^{ini}$ is the initial SC voltage. The Simulink model of the SC is shown in Fig 8.

The use of both a battery (high energy density) and an SC (high power density) achieve a satisfactory driving range while meeting transient power demands at a suitable cost [40]. The SC reduces the battery stress by delivering power to the transients during harsh accelerations and stores regenerative energy in aggressive decelerations [39]. Important SC parameters are given in Table 6.

**Table 6:** Parameters of SC.

Supercapacitor	
**Type**	Maxwell Boost Cap BMOD0165-48.6 VUC
**Capacitance**	165 F
**Number of series capacitors**	50
**Number of parallel capacitors**	20
**Number of module**	12
**Rated voltage**	12 × 48:6 ≈ 584 V

#### 2.1.7 Micro-turbine

**Fig 9:**
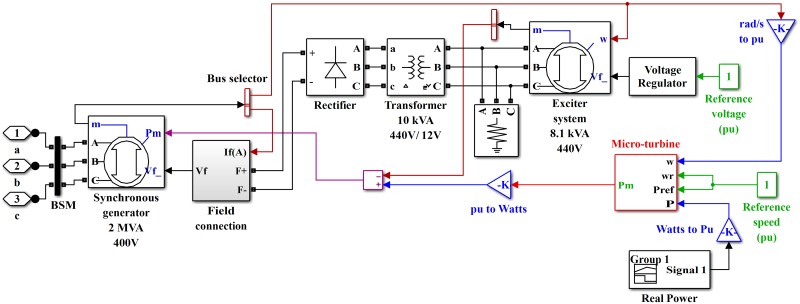
Simulink model of split-shaft MT with synchronous generator.

The MT is basically a smaller version of a heavy-duty gas turbine, deploying a radial compressor, combustor and turbine rotor with 25–300 kW installed capacity. The speed and power of the MT under dynamic load conditions are controlled using PI controllers, which work on the error signals. The fuel system involves the valve positioner and actuator. The temperature is controlled to obtain the mechanical power at a predetermined firing temperature. The MT generates electrical power though the synchronous generator. The turbine torque can be calculated as follows:

$$\tau_{turbine}=1.3\left(f_{d}-0.23\right)+0.5(1-\omega )$$ (19)

where *τ*_*turbine*_ is the torque of the turbine, *f*_*d*_ is the fuel demand, and *ω* is the per unit speed of the turbine. The net mechanical power generated by the MT is

$$P_{mt}(t)=P_{turbine}(t)-P_{compressor}(\text{t})$$ (20)

where *P*_*mt*_ is the net mechanical power produced by the MT, *P*_*turbine*_ is the power produced by the turbine, and *P*_*compressor*_ is the power consumed by the compressor. *P*_*mt*_ is exerted on the turbine shaft to obtain the net electrical power at the output terminals of the MT as follows:

$$P_{mt}^{gen}(t)=P_{mt}(t)\eta_{m}\eta_{g}\eta_{e}$$ (21)

where $P_{mt}^{gen}$ is the generated electrical power of the MT, *η*_*m*_ is the mechanical efficiency, *η*_*g*_ is the generator efficiency, and *η*_*e*_ is the electrical efficiency for interfacing. A split-shaft MT system with a two-pole synchronous generator is shown in Fig 9.

**Fig 10:**
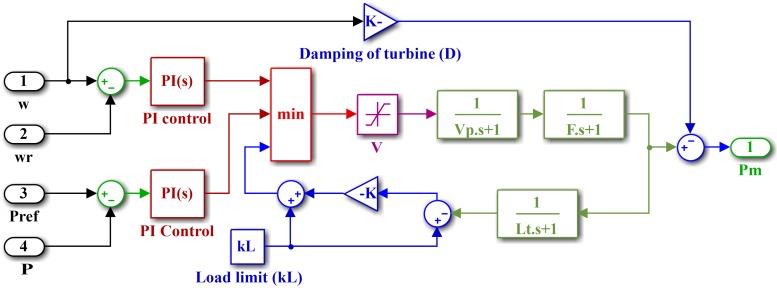
Simulink model of micro-turbine.

The details of the MT model are shown in Fig 10. The parameters of the MT are listed in Table 7.

**Table 7:** Parameters of micro-turbine.

Micro-turbine	
**Type**	Ingersoll Rand MT250
**Rated power**	200 kVA, 160 kW
**Rated voltage**	440 V
**Rated frequency**	50 Hz

## 3. Proposed adaptive control paradigm

### 3.1 Indirect adaptive MPPT control of PV system

The PV MPPT is used to move the operating voltage under varying atmospheric conditions to maintain its position at the MPP. When the change of PV power with respect to the operating voltage is zero, the MPP is achieved as follows:

$$s=\frac{\partial P_{pv}}{\partial V_{pv}}=\frac{I_{pv}}{V_{pv}}+\frac{\partial I_{pv}}{\partial V_{pv}}=0$$ (22)

where *s* is the slope of PV power with respect to the operating voltage. Eq 22 is solved to calculate the MPP voltage at each instant of time using the MPPT algorithm. A Hermite wavelet embedded NeuroFuzzy indirect adaptive controller (HWNFIAC) is used to extract the maximum PV power. In the proposed control system, the incorporation of the Hermite wavelet embedded NeuroFuzzy identifier (HWNFI) makes it indirect. Both the controller and identifier are based on the same NeuroFuzzy structure.

#### 3.1.1 Hermite wavelet embedded NeuroFuzzy identifier for PV

The Hermite wavelet has a restriction-free input range, which makes it more appropriate for solving highly nonlinear problems with a wide search space [41], [42]. Moreover, the series expansion of sufficient Hermite polynomials is used to represent any signal with a high degree of accuracy. The recursive relationships of Hermite polynomials and their first-order derivatives are efficiently used in the constructive network design.

The Hermite polynomial *H*_*m*_(*x*) of order *m* is defined on the interval [−∞, ∞] and is given as

$$H_{0}(\text{x})=1,\;H_{1}(\text{x})=2x\text{ and }H_{m+1}(\text{x})=2xH_{m}(\text{x})-2mH_{m-1}(\text{x})$$ (23)

where *H*_*m*_(*x*) is orthogonal with respect to the weight function as

$$\int_{-\infty }^{\infty }e^{-x^{2}}H_{m}(\text{x})H_{n}(\text{x})=\{\begin{array}{c} 0,\\ n!2^{n}\sqrt{\pi }, \end{array}\;\begin{array}{c} m\neq n\\ m=n \end{array}$$ (24)

Hermite wavelet *ψ*_*n*_,_*m*_(*x*) is defined on the interval [0,1) by

$$\psi_{n,m}(x)=\{\begin{array}{c} 2^{k/2}\sqrt{\frac{1}{n!2^{n}\sqrt{\pi }}}H_{m}(2^{k}x-\overset{⌢}{\text{n}}),\\ 0, \end{array}\;\begin{array}{c} \frac{\overset{⌢}{\text{n}}-1}{2^{k}}\leq x\leq \frac{\overset{⌢}{\text{n}}-1}{2^{k}}\\ \text{Otherwise} \end{array}$$ (25)

where *k* = 1, 2, ⋯, is the level of resolution, *n* = 1, 2, ⋯, 2^*k*-1^, $\overset{⌢}{n}=2n-1$, is the translation parameter, and *m* = 1, 2, ⋯, *M* − 1 is the order of the polynomial, *M* > 0.

**Fig 11:**
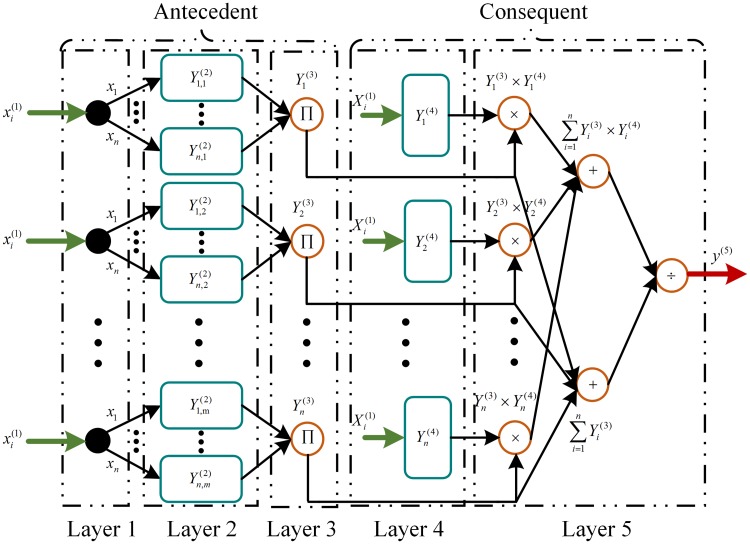
NeuroFuzzy network.

The NeuroFuzzy network is based on a five-layer feedforward connectionist network as shown in Fig 11.

1. *Layer 1* is the input layer, which directly transmits the inputs to the second layer. The input $x_{i}^{\left(1\right)}$ and the output $y_{i,j}^{\left(1\right)}$ for this layer are given as
  $$\{\begin{array}{c} x_{i}^{\left(1\right)}=x_{i},\\ y_{i,j}^{\left(1\right)}=x_{i}^{\left(1\right)}, \end{array}\;\begin{array}{c} i=1,\;2,\;\cdots ,\text{ n}\\ j=1,\;2,\;\cdots ,\;m \end{array}$$ (26)
2. *Layer 2* is the fuzzification layer, which uses a Gaussian membership function to fuzzify the inputs of the antecedent part as follows:
  $$\{\begin{array}{c} x_{i,j}^{\left(2\right)}=-\frac{{\left(y_{i,j}^{\left(1\right)}-c_{i,j}\right)}^{2}}{s_{i,j}^{2}}\\ y_{i,j}^{\left(2\right)}=\text{exp}\left(x_{i,j}^{\left(2\right)}\right) \end{array}\;$$ (27)
  where *c*_*i*,*j*_ and *s*_*i*,*j*_ represent the center and spread of the Gaussian membership function, respectively.
3. *Layer 3* is the rule layer, which uses the product T-norm to compute the firing strength of each rule as follows:
  $$\{\begin{array}{c} x_{\text{i}}^{\left(3\right)}=\prod y_{\text{i},j}^{\left(2\right)}\\ y_{i}^{\left(3\right)}=x_{i}^{\left(3\right)} \end{array}\;$$ (28)
4. *Layer 4* is the consequent layer, which presents the Hermite wavelet function in each node. The weighted consequent value is given by
  $$\{\begin{array}{c} x_{i}^{\left(4\right)}=\psi_{i,j}(x_{i})\\ y_{i}^{\left(4\right)}=\sum_{i=1}^{2^{k-1}}w_{i}x_{i}^{\left(4\right)} \end{array}\;$$ (29)
5. *Layer 5* is the output layer, which defuzzifies the network output as follows:
  $$\{\begin{array}{c} x_{i}^{\left(5\right)}=\sum_{i=1}^{n}y_{i}^{\left(3\right)}y_{i}^{\left(4\right)}\\ y_{i}^{\left(5\right)}=\frac{x_{i}^{\left(5\right)}}{\sum_{i=1}^{n}y_{i}^{\left(3\right)}} \end{array}\;$$ (30)
  where $y_{i}^{\left(5\right)}\in \{\hat{\text{s}},{\text{u}}_{pv}\}$ is the output of the Hermite wavelet embedded NeuroFuzzy network such that $\hat{\text{s}}$ is the output of the HWNFI and *u*_*pv*_ is the output of the HWNFIAC. The error function used to adjust the HWNFI parameters is given as
  $$e_{I}=\left(\hat{s}(\text{t})-s(\text{t})\right)$$ (31)
  where *s*(t) is the PV plant output, and $\hat{s}(\text{t})$ is the HWNFI output. The gradient descent algorithm is employed to adjust the linking weight *w*_*i*_ and parameters of the Gaussian membership function—i.e., *c*_*i*,*j*_ and *ss*_*i*,*j*_. Thus, the generalized parameter update law is written as
  $$\xi_{i,j}(\text{t}+1)=\xi_{i,j}(\text{t})+\eta e_{I}(\text{t})\frac{\partial \hat{s}(\text{t})}{\partial \xi_{i,j}(\text{t})}$$ (32)
  where ξ_*i*,*j*_ ∈ {w_*i*,*j*_, *c*_*i*,*j*_, *ss*_*i*,*j*_}, and *η* is the learning rate. The differential term $\frac{\partial \hat{s}(\text{t})}{\partial \xi_{i,j}(\text{t})}$ is simplified for respective parameters by applying the chain rule as follows:
  $$\frac{\partial \hat{s}}{\partial w_{i,j}}=\frac{\partial \hat{s}}{\partial y_{i}^{\left(4\right)}}\frac{\partial y_{i}^{\left(4\right)}}{\partial w_{i,j}}$$ (33)
  $$\frac{\partial \hat{s}}{\partial c_{i,j}}=\frac{\partial \hat{s}}{\partial y_{i}^{\left(3\right)}}\frac{\partial y_{i}^{\left(3\right)}}{\partial y_{i,j}^{\left(2\right)}}\frac{\partial y_{i,j}^{\left(2\right)}}{\partial c_{i,j}}$$ (34)
  $$\frac{\partial \hat{s}}{\partial ss_{i,j}}=\frac{\partial \hat{s}}{\partial y_{i}^{\left(3\right)}}\frac{\partial y_{i}^{\left(3\right)}}{\partial y_{i,j}^{\left(2\right)}}\frac{\partial y_{i,j}^{\left(2\right)}}{\partial ss_{i,j}}$$ (35)

After simplifying the above differentials for each parameter, the final updated equations are given as follows:

$$w_{i,j}(\text{t}+1)=w_{i,j}(\text{t})+\eta (\hat{s}(\text{t})-s(\text{t}))\left[\frac{y_{i}^{\left(3\right)}}{\sum_{i=1}^{n}y_{i}^{\left(3\right)}}x_{i}^{\left(4\right)}\right]$$ (36)

$$c_{i,j}(\text{t}+1)=c_{i,j}(\text{t})+\eta (\hat{s}(\text{t})-s(\text{t}))\left[\frac{y_{i}^{\left(4\right)}-\hat{s}(\text{t})}{\sum_{i=1}^{n}y_{i}^{\left(3\right)}}\right]y_{i}^{\left(3\right)}\frac{\left(x_{i}-c_{i,j}\right)}{ss_{i,j}^{2}}$$ (37)

$$ss_{i,j}(\text{t}+1)=ss_{i,j}(\text{t})+\eta (\hat{s}(\text{t})-s(\text{t}))\left[\frac{y_{i}^{\left(4\right)}-\hat{s}(\text{t})}{\sum_{i=1}^{n}y_{i}^{\left(3\right)}}\right]y_{i}^{\left(3\right)}\frac{{\left(x_{i}-c_{i,j}\right)}^{2}}{ss_{i,j}^{3}}$$ (38)

#### 3.1.2 Hermite wavelet embedded NeuroFuzzy indirect adaptive controller for PV

The controller uses the same Hermite wavelet embedded NeuroFuzzy structure as used by the HWNFI. The error function to adjust the HWNFIAC parameters is given as

$$e_{c}(\text{t})=\left(\text{r}(\text{t})-s(\text{t})\right)$$ (39)

where *r*(t) is the reference input. The parameters of the HWNFIAC are updated by minimizing the following cost function:

$$\Phi_{c}=\frac{1}{2}\left[e_{c}^{2}(\text{t})+\hbar u_{pv}^{2}(\text{t})\right]$$ (40)

where Φ_*c*_ is the cost function, and ℏ is the learning rate. Thus, the generalized adaptive law can be written as

$${ℑ}_{i,j}(\text{t}+1)={ℑ}_{i,j}(\text{t})+\hbar \frac{\partial \Phi_{c}(\text{t})}{\partial {ℑ}_{i,j}(\text{t})}+\hbar \Delta {\text{e}}_{c}(t)$$ (41)

where ℑ ∈ {*v*_*i*,*j*_, *k*_*i*,*j*_, *σ*_*i*,*j*_} is the adaptation vector for the HWNFIAC, which can be calculated by using the gradient descent algorithm. The term $\frac{\partial \Phi_{c}(\text{t})}{\partial {ℑ}_{i,j}(\text{t})}$ can be simplified using the following equation:

$$\frac{\partial \Phi_{c}(\text{t})}{\partial {ℑ}_{i,j}(\text{t})}=\left[e_{c}(\text{t})\frac{\partial \hat{s}}{\partial u_{pv}}-\hbar u_{pv}\right]\frac{\partial u_{pv}}{\partial {ℑ}_{i,j}(\text{t})}$$ (42)

The term $\frac{\partial \hat{s}}{\partial u_{pv}}$ is based on the HWNFI and can be calculated as follows:

$$\frac{\partial \hat{s}}{\partial u_{pv}}=\frac{\sum_{i=1}^{n}y_{i}^{\left(3\right)}\left[-\left(\frac{u_{pv}-c_{i,j}}{ss_{i,j}^{2}}\right)y_{i}^{\left(4\right)}-\hat{s}(\text{t})+2\sqrt{\frac{2}{\pi }}\left\{8\varphi_{11}^{j}+\varphi_{12}^{j}(128{\text{u}}_{pv}-\chi )\right\}\right]}{\sum_{i=1}^{n}y_{i}^{\left(3\right)}}$$ (43)

where *χ* is given as

$$\{\begin{array}{c} \chi =32,\\ \chi =48, \end{array}\;\begin{array}{c} 0\leq u_{pv}\leq 1/2\\ 1/2\leq u_{pv}\leq 1 \end{array}$$ (44)

The differential term $\frac{\partial u_{pv}(\text{t})}{\partial {ℑ}_{i,j}(\text{t})}$ from Eq 42 is simplified for the respective parameters by applying the chain rule as follows:

$$\frac{\partial u_{pv}}{\partial \nu_{i,j}}=\frac{\partial u_{pv}}{\partial y_{i}^{\left(4\right)}}\frac{\partial y_{i}^{\left(4\right)}}{\partial \nu_{i,j}}$$ (45)

$$\frac{\partial u_{pv}}{\partial \kappa_{i,j}}=\frac{\partial u_{pv}}{\partial y_{i}^{\left(3\right)}}\frac{\partial y_{i}^{\left(3\right)}}{\partial y_{i,j}^{\left(2\right)}}\frac{\partial y_{i,j}^{\left(2\right)}}{\partial \kappa_{i,j}}$$ (46)

$$\frac{\partial u_{pv}}{\partial \sigma_{i,j}}=\frac{\partial u_{pv}}{\partial y_{i}^{\left(3\right)}}\frac{\partial y_{i}^{\left(3\right)}}{\partial y_{i,j}^{\left(2\right)}}\frac{\partial y_{i,j}^{\left(2\right)}}{\partial \sigma_{i,j}}$$ (47)

After simplifying the above differentials for each parameter, the final update equations for the HWNFIAC are given as follows:

$$\nu_{i,j}(\text{t}+1)=\nu_{i,j}(\text{t})+\hbar \left(\left(r(\text{t})-s(\text{t})\right)\frac{\partial \hat{s}}{\partial u_{pv}}-\hbar u_{pv}\right)\left[\frac{y_{i}^{\left(3\right)}}{\sum_{i=1}^{n}y_{i}^{\left(3\right)}}x_{i}^{\left(4\right)}\right]$$ (48)

$$\kappa_{i,j}(\text{t}+1)=\kappa_{i,j}(\text{t})+\hbar \left(\left(r(\text{t})-s(\text{t})\right)\frac{\partial \hat{s}}{\partial u_{pv}}-\hbar u_{pv}\right)\left[\frac{y_{i}^{\left(4\right)}-u_{pv}(\text{t})}{\sum_{i=1}^{n}y_{i}^{\left(3\right)}}\right]y_{i}^{\left(3\right)}\frac{\left(x_{i}-\kappa_{i,j}\right)}{\sigma_{i,j}^{2}}$$ (49)

where *x*_*i*_ = *e*_*c*_(t) or *x*_*i*_ = Δ*e*_*c*_(t).

$$\sigma_{i,j}(\text{t}+1)=\sigma_{i,j}(\text{t})+\hbar \left(\left(r(\text{t})-s(\text{t})\right)\frac{\partial \hat{s}}{\partial u_{pv}}-\hbar u_{pv}\right)\left[\frac{y_{i}^{\left(4\right)}-u_{pv}(\text{t})}{\sum_{i=1}^{n}y_{i}^{\left(3\right)}}\right]y_{i}^{\left(3\right)}\frac{{\left(x_{i}-\kappa_{i,j}\right)}^{2}}{\sigma_{i,j}^{3}}$$ (50)

### 3.2 Indirect adaptive control of SOFC system

To obtain the swift response from the SOFC, the input hydrogen must be controlled. The input hydrogen is directly proportional to the SOFC stack current. Therefore, the optimal flow of input hydrogen is obtained by controlling the SOFC stack current. The relationship for the SOFC stack current is given by

$$m_{H_{2}}^{in}=\left(\frac{2k}{H_{2}^{uti}}\right)I_{sofc}\Rightarrow I_{sofc}=\left(\frac{H_{2}^{uti}}{2k}\right)m_{H_{2}}^{in}$$ (51)

where $H_{2}^{uti}$ is the optimal hydrogen utilization, and $m_{H_{2}}^{in}$ is the molar flow of input hydrogen. $H_{2}^{uti}$ has a typical range of 80–90%. For optimal hydrogen utilization, the SOFC current lies in the following limits:

$$\frac{0.8m_{H_{2}}^{in}}{2k}=I_{sofc-\text{min}}\leq I_{sofc-r}\leq I_{sofc-\text{max}}=\frac{0.9m_{H_{2}}^{in}}{2k}$$ (52)

where *k* is the constant that gives the amount of hydrogen reacting in the SOFC, and $0.8m_{H_{2}}^{in}$ and $0.9m_{H_{2}}^{in}$ are the minimum and maximum limits of molar flow of hydrogen, respectively. I_*sofc*−min_, I_*sofc*−r_ and I_*sofc*−max_ are the minimum, reference and maximum SOFC currents, respectively. The aforementioned limitations of hydrogen utilization and current helps the SOFC acquire the optimal operating point of the V–I curve, because the output power of the SOFC is directly related to its fuel consumption. The load variations suggest the different output power levels for the SOFC. Different SOFC output power levels require appropriate variation in the input hydrogen flowrate, which is possible by a control system. The SOFC power demand is converted into current as follows:

$$I_{sofc-r}=\frac{P_{sofc-r}}{V_{sofc}}$$ (53)

A Hermite wavelet incorporated NeuroFuzzy indirect adaptive control is used to obtain the swift response from the SOFC. To identify the SOFC plant, a Hermite wavelet embedded NeuroFuzzy identifier is used. In both the controller and identifier, Hermite wavelets are embedded in the consequent part of the NeuroFuzzy network. The structure of the Hermite wavelet incorporated NeuroFuzzy indirect adaptive control for SOFC is the same as that used for the PV system.

#### 3.2.1 Hermite wavelet embedded NeuroFuzzy identifier for SOFC

The output of the Hermite wavelet embedded NeuroFuzzy network is $y_{i}^{\left(5\right)}\in \{{\hat{\text{I}}}_{sofc},{\text{u}}_{sofc}\}$, where ${\hat{\text{I}}}_{sofc}$ is the output current of the HWNFI, and *u*_*sofc*_ is the output of the controller. The error function ${\tilde{e}}_{I}$ to adjust the HWNFI parameters is given as

$${\tilde{e}}_{I}(\text{t})=\left({\hat{\text{I}}}_{sofc}(\text{t})-{\text{I}}_{sofc}(\text{t})\right)$$ (54)

where I_*sofc*_(t) is the SOFC plant output, and ${\hat{\text{I}}}_{sofc}(\text{t})$ is the HWNFI output. The gradient descent algorithm is employed to adjust the linking weight ${\tilde{w}}_{i}$, center ${\tilde{c}}_{i,j}$ and spread $\tilde{s}{\tilde{s}}_{i,j}$ of the Gaussian membership function. Therefore, the generalized parameters update law is written as

$${\tilde{\xi }}_{i,j}(\text{t}+1)={\tilde{\xi }}_{i,j}(\text{t})+\tilde{\eta }{\tilde{e}}_{I}(\text{t})\frac{\partial {\hat{I}}_{sofc}(\text{t})}{\partial {\tilde{\xi }}_{i,j}(\text{t})}$$ (55)

where ${\tilde{\xi }}_{i,j}\in \{{\tilde{w}}_{i,j},\;{\tilde{c}}_{i,j},\;\tilde{s}{\tilde{s}}_{i,j}\}$, and $\tilde{\eta }$ is the learning rate for the HWNFI. After simplifying the differential $\frac{\partial {\hat{I}}_{sofc}(\text{t})}{\partial {\tilde{\xi }}_{i,j}(\text{t})}$ for each parameter, the final updated equations are as follows:

$${\tilde{w}}_{i,j}(\text{t}+1)={\tilde{w}}_{i,j}(\text{t})+\tilde{\eta }({\hat{\text{I}}}_{sofc}(\text{t})-{\text{I}}_{sofc}(\text{t}))\left[\frac{y_{i}^{\left(3\right)}}{\sum_{i=1}^{n}y_{i}^{\left(3\right)}}\vartheta_{n,m}(x)\right]$$ (56)

$${\tilde{c}}_{i,j}(\text{t}+1)={\tilde{c}}_{i,j}(\text{t})+\tilde{\eta }({\hat{\text{I}}}_{sofc}(\text{t})-{\text{I}}_{sofc}(\text{t}))\left[\frac{y_{i}^{\left(4\right)}-{\hat{\text{I}}}_{sofc}(\text{t})}{\sum_{i=1}^{n}y_{i}^{\left(3\right)}}\right]y_{i}^{\left(3\right)}\frac{\left(x_{i}-{\tilde{c}}_{i,j}\right)}{\tilde{s}{\tilde{s}}_{i,j}^{2}}$$ (57)

$$\tilde{s}{\tilde{s}}_{i,j}(\text{t}+1)=\tilde{s}{\tilde{s}}_{i,j}(\text{t})+\tilde{\eta }({\hat{\text{I}}}_{sofc}(\text{t})-{\text{I}}_{sofc}(\text{t}))\left[\frac{y_{i}^{\left(4\right)}-{\hat{\text{I}}}_{sofc}(\text{t})}{\sum_{i=1}^{n}y_{i}^{\left(3\right)}}\right]y_{i}^{\left(3\right)}\frac{{\left(x_{i}-{\tilde{c}}_{i,j}\right)}^{2}}{\tilde{s}{\tilde{s}}_{i,j}^{3}}$$ (58)

#### 3.2.2 Hermite wavelet embedded NeuroFuzzy indirect adaptive controller for SOFC

The HWNFIAC uses the same Hermite wavelet embedded NeuroFuzzy structure as used by the HWNFI. The error function to adjust the HWNFIAC parameters is given as

$${\tilde{e}}_{c}(\text{t})=\left(\tilde{\text{r}}(\text{t})-I_{sofc}(\text{t})\right)$$ (59)

where $\tilde{r}(\text{t})$ is the reference input. To update the parameters of the HWNFIAC, the following cost function is minimized:

$${\tilde{\Phi }}_{c}=\frac{1}{2}\left[{\tilde{e}}_{c}^{2}(\text{t})+\tilde{\hbar }u_{sofc}^{2}(\text{t})\right]$$ (60)

where ${\tilde{\Phi }}_{c}$ is the cost function, and $\tilde{\hbar }$ is the learning rate for the HWNFIAC. Thus, the generalized adaptive law is written as

$${\tilde{ℑ}}_{i,j}(\text{t}+1)={\tilde{ℑ}}_{i,j}(\text{t})+\tilde{\hbar }\frac{\partial {\tilde{\Phi }}_{c}(\text{t})}{\partial {\tilde{ℑ}}_{i,j}(\text{t})}+\tilde{\hbar }\Delta {\tilde{\text{e}}}_{c}(\text{t})$$ (61)

where the adaptation vector $\tilde{ℑ}\in \left\{{\tilde{\nu }}_{i,j},\;{\tilde{\kappa }}_{i,j},\;{\tilde{\sigma }}_{i,j}\right\}$ for the HWNFIAC can be calculated by using the gradient descent algorithm. The term $\frac{\partial {\tilde{\Phi }}_{c}(\text{t})}{\partial {\tilde{ℑ}}_{i,j}(\text{t})}$ can be simplified using the following equation:

$$\frac{\partial {\tilde{\Phi }}_{c}(\text{t})}{\partial {\tilde{ℑ}}_{i,j}(\text{t})}=\left[{\tilde{e}}_{c}(\text{t})\frac{\partial I_{sofc}}{\partial u_{sofc}}-\hbar u_{sofc}\right]\frac{\partial u_{sofc}}{\partial {\tilde{ℑ}}_{i,j}(\text{t})}$$ (62)

The term $\frac{\partial {\hat{I}}_{sofc}}{\partial u_{sofc}}$ is based on the HWNFI and can be calculated as follows:

$$\frac{\partial {\hat{I}}_{sofc}}{\partial u_{sofc}}=\frac{\sum_{i=1}^{n}y_{i}^{\left(3\right)}\left[-\left(\frac{u_{sofc}-{\tilde{c}}_{i,j}}{\tilde{s}{\tilde{s}}_{i,j}^{2}}\right)y_{i}^{\left(4\right)}-{\hat{I}}_{sofc}(\text{t})+2\sqrt{\frac{2}{\pi }}\left\{8{\tilde{\varphi }}_{11}^{j}+{\tilde{\varphi }}_{12}^{j}(128{\text{u}}_{sofc}-\tilde{\chi })\right\}\right]}{\sum_{i=1}^{n}y_{i}^{\left(3\right)}}$$ (63)

where $\tilde{\chi }$ is given as

$$\{\begin{array}{c} \tilde{\chi }=32,\\ \tilde{\chi }=48, \end{array}\;\begin{array}{c} 0\leq u_{sofc}\leq 1/2\\ 1/2\leq u_{sofc}\leq 1 \end{array}$$ (64)

The differential term $\frac{\partial u_{sofc}(\text{t})}{\partial {\tilde{ℑ}}_{i,j}(\text{t})}$ from Eq 62 is simplified for the respective parameters by applying the chain rule as follows:

$$\frac{\partial u_{sofc}}{\partial {\tilde{\nu }}_{i,j}}=\frac{\partial u_{sofc}}{\partial y_{i}^{\left(4\right)}}\frac{\partial y_{i}^{\left(4\right)}}{\partial {\tilde{\nu }}_{i,j}}$$ (65)

$$\frac{\partial u_{sofc}}{\partial {\tilde{\kappa }}_{i,j}}=\frac{\partial u_{sofc}}{\partial y_{i}^{\left(3\right)}}\frac{\partial y_{i}^{\left(3\right)}}{\partial y_{i,j}^{\left(2\right)}}\frac{\partial y_{i,j}^{\left(2\right)}}{\partial {\tilde{\kappa }}_{i,j}}$$ (66)

$$\frac{\partial u_{pv}}{\partial \sigma_{i,j}}=\frac{\partial u_{pv}}{\partial y_{i}^{\left(3\right)}}\frac{\partial y_{i}^{\left(3\right)}}{\partial y_{i,j}^{\left(2\right)}}\frac{\partial y_{i,j}^{\left(2\right)}}{\partial \sigma_{i,j}}$$ (67)

After simplifying the above differentials for each parameter, the final update equations for the HWNFIAC are given as follows:

$${\tilde{\nu }}_{i,j}(\text{t}+1)={\tilde{\nu }}_{i,j}(\text{t})+\tilde{\hbar }\left(\left(\tilde{r}(\text{t})-I_{sofc}(\text{t})\right)\frac{\partial {\hat{I}}_{sofc}}{\partial u_{sofc}}-\tilde{\hbar }u_{sofc}\right)\left[\frac{y_{i}^{\left(3\right)}}{\sum_{i=1}^{n}y_{i}^{\left(3\right)}}x_{i}^{\left(4\right)}\right]$$ (68)

$${\tilde{\kappa }}_{i,j}(\text{t}+1)={\tilde{\kappa }}_{i,j}(\text{t})+\tilde{\hbar }\left(\left(\tilde{r}(\text{t})-I_{sofc}(\text{t})\right)\frac{\partial {\hat{I}}_{sofc}}{\partial u_{sofc}}-\tilde{\hbar }u_{sofc}\right)\left[\frac{y_{i}^{\left(4\right)}-u_{sofc}(\text{t})}{\sum_{i=1}^{n}y_{i}^{\left(3\right)}}\right]y_{i}^{\left(3\right)}\frac{\left(x_{i}-{\tilde{\kappa }}_{i,j}\right)}{{\tilde{\sigma }}_{i,j}^{2}}$$ (69)

where $x_{i}={\tilde{e}}_{c}(\text{t})$ or $x_{i}=\Delta {\tilde{e}}_{c}(\text{t})$.

$${\tilde{\sigma }}_{i,j}(\text{t}+1)={\tilde{\sigma }}_{i,j}(\text{k})+\tilde{\hbar }\left(\left(\tilde{r}(\text{t})-I_{sofc}(\text{t})\right)\frac{\partial {\hat{I}}_{sofc}}{\partial u_{sofc}}-\tilde{\hbar }u_{sofc}\right)\left[\frac{y_{i}^{\left(4\right)}-u_{sofc}(\text{t})}{\sum_{i=1}^{n}y_{i}^{\left(3\right)}}\right]y_{i}^{\left(3\right)}\frac{{\left(x_{i}-{\tilde{\kappa }}_{i,j}\right)}^{2}}{{\tilde{\sigma }}_{i,j}^{3}}$$ (70)

Eqs 22 and 52 are solved to compute the voltage at the MPP for the PV system and the swift response of the SOFC at each instant of time subject to the following assumptions:

$$Assumption\;1:\;\{\begin{array}{c} THD_{V}<5\%,\;THD_{I}<5\%,\\ -0.8\%f_{fund}<f_{fund}<+0.8\%f_{fund}\\ -6\%{\text{V}}_{rms}<{\text{V}}_{rms}<+6\%{\text{V}}_{rms}\\ -5\%{\text{V}}_{DC\;bus}<{\text{V}}_{DC\;bus}<+5\%{\text{V}}_{DC\;bus} \end{array}$$ (71)

&

$$Assumption\;2:\;\{\begin{array}{c} P_{gen}+P_{mt}\pm P_{grid}=P_{load}\\ Q_{gen}+Q_{mt}\pm Q_{grid}=Q_{load} \end{array}$$ (72)

where *THD*_*V*_ and *THD*_*I*_ are the total harmonic distortion for the load voltage and current, respectively. *f*_*fund*_ is the fundamental frequency of the load in which only 0.8% fluctuations are allowed to obtain the quality power. *V*_*rms*_ is load rms voltage, and the acceptable variation in *V*_*rms*_ is up to 6%. V_*DC bus*_ is the DC bus voltage variation, which should remain constant for stable operation of the HPS. *P*_*gen*_ and *Q*_*gen*_ are the active and reactive generated power from the renewable energy sources along with the backup system. *P*_*mt*_ and *Q*_*mt*_ are the active and reactive power of the MT. *P*_*grid*_ and *Q*_*grid*_ are the active and reactive powers of the utility grid. A ± symbol with grid powers shows the bidirectional flow of grid powers. *P*_*load*_ and *Q*_*load*_ are the active and reactive powers of the load. The power from renewable energy sources is calculated as follows:

$$P_{gen}=P_{wind}+P_{pv}\pm P_{sc}\pm P_{bat}+P_{sofc}-P_{elect}$$ (73)

where *P*_*sc*_, *P*_*bat*_ and *P*_*elect*_ are the output powers of the SC, battery and electrolyzer, respectively. The following closed-loop control algorithm steps are used to update the parameters of the identifiers and controllers of the PV and SOFC.

1. Initialize the linking weights and parameters of Gaussian membership functions of identifiers and controllers.
2. Adjust the values of learning rates.
3. Sample the inputs of the HWNFIAC at time *t*.
4. Update the parameters of the HWNFI by minimizing the respective error—i.e., *e*_*I*_ (t) and ${\tilde{e}}_{I}(\text{t})$.
5. Calculate the outputs of the controllers (*u*_*pv*_ and *u*_*sofc*_) and apply them to the respective plant.
6. Compute the outputs of PV and SOFC plants using control signals.
7. Calculate the adaptation errors using $\hat{s}$ and ${\hat{I}}_{sofc}$. Back propagate these errors to adjust the parameters of HWNFIAC by minimizing Φ_*c*_ and ${\tilde{\Phi }}_{c}$.
8. Repeat steps 2–7 until the solution converges.

**Fig 12:**
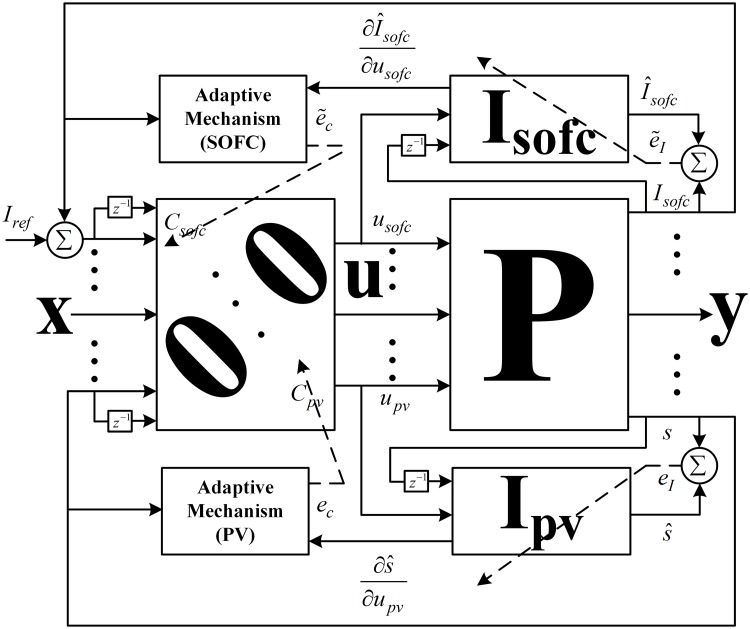
Closed-loop control systems for PV and SOFC.

The closed-loop PV and SOFC control systems are shown in Fig 12.

### 3.3 Supervisory control

**Fig 13:**
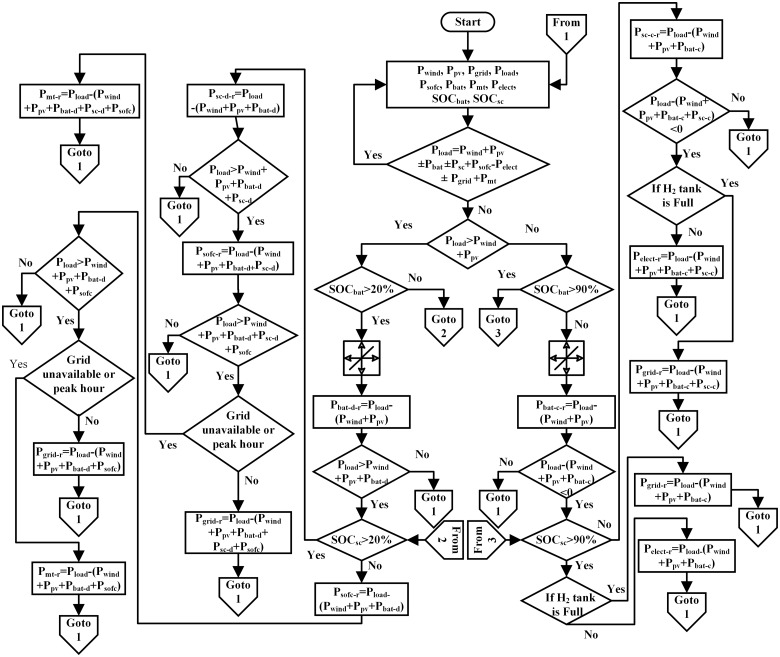
HPS supervisory control flowchart.

The prime obligation of the supervisory control is to ensure the continuous supply of power in the HPS. The supervisory control calculates the reference power for the SOFC, electrolyzer, battery, SC, MT and grid based on the deduction of the load power from the wind and PV generated power. The flowchart of supervisory control is shown in Fig 13. In the flowchart, *P*_*wind*_, *P*_*pv*_, *P*_*sofc*_, *P*_*elect*_, *P*_*bat*_, *P*_*sc*_, *P*_*grid*_ and *P*_*mt*_ are the wind, PV, SOFC, electrolyzer, battery, SC, grid and MT power, respectively. *SOC*_*bat*_ and *SOC*_*sc*_ are the SOCs of the battery and SC, respectively. Supervisory control requires several decisions for the management and use of power. The decision factor of the supervisory control depends upon the load power and the wind and PV generated power. The SOFC, battery, SC, MT and grid are capable of providing the required power. All steps for the operation of supervisory control are explained below:

1. Compare the wind and PV generated power with the load. If the wind and PV generated power is greater than the load, go to step 2; otherwise, go to step 6.
2. If the SOC of the battery is less than 90%, the battery is in charge mode. If the battery is charged and the generation (wind and PV power) is greater than the load, go to step 3; otherwise, go to step 1.
3. If the SOC of the SC is less than 90%, the SC is in charge mode. If the SC is charged and the generation (wind and PV power) is greater than the load, go to step 4; otherwise, go to step 1.
4. If both the battery and SC are fully charged, the excess power is used by the electrolyzer to produce the hydrogen gas. If the hydrogen tank is full and the generation (wind and PV power) is greater than the load, go to step 5; otherwise, go to step 1.
5. The excess power is given to the grid; go to step 1.
6. If the wind and PV generated power is less than the load power and the SOC of the battery is greater than 20%, the battery is in discharge mode. If the battery is discharged and deficient power exists, go to step 7; otherwise, go to step 1.
7. If the SOC of the SC is greater than 20%, the SC is in discharge mode. If the SC is discharged and deficient power exists, go to step 8; otherwise, go to step 1.
8. If both the battery and SC are fully discharged, the SOFC delivers the power to the load. If the load is not met, go to step 9. Otherwise, go to step 1.
9. The grid delivers the remaining deficient power if it is available or during off-peak hours; otherwise, the MT delivers the remaining deficient power. Go to step 1.

## 4. Results and discussions

**Fig 14:**
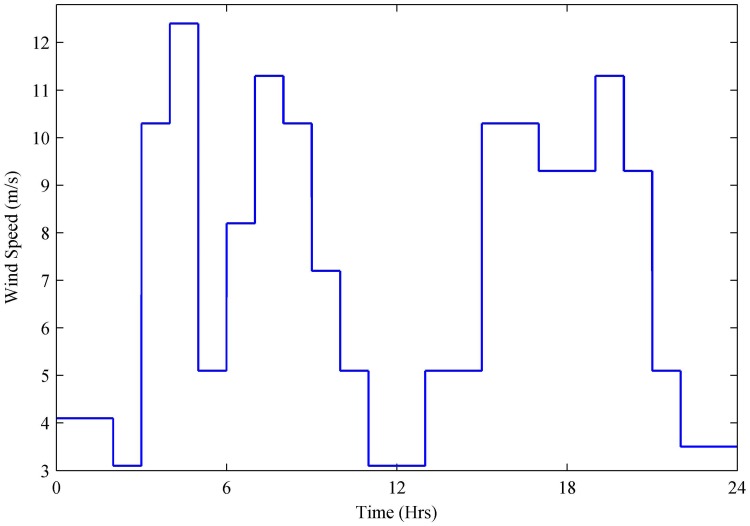
Wind speed.

**Fig 15:**
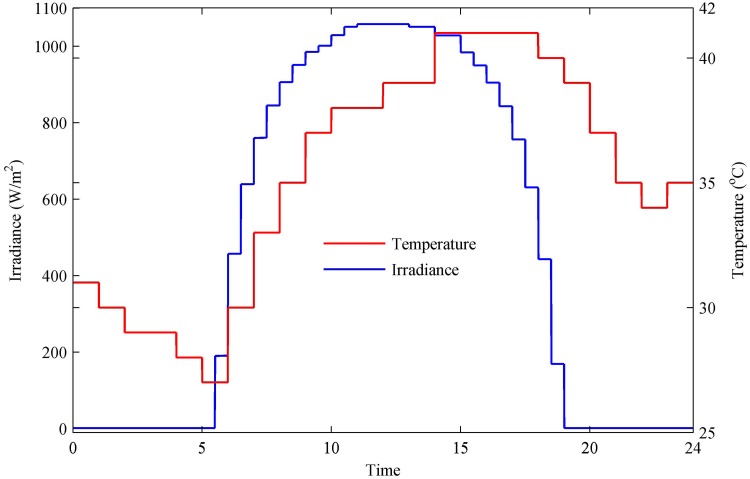
Temperature and irradiance levels.

The performance of the stated HPS and proposed controllers is evaluated in MATLAB Simulink R2014b. In an 11 kV grid-connected HPS, wind generation of 100 kW, PV of 260 kW, SOFC of 200 kW, electrolyzer of 150 kW, and MT of 200 kVA along with backup sources (200 Ah battery and 165 F Super-Capacitor) are modeled for the dynamic residential load. Defense Housing Authority (DHA), Islamabad, Pakistan, is taken as a case study. The hourly basis wind speed (m/s), irradiance (W/m^2^) and ambient temperature (°C) levels are recorded by the Pakistan Meteorological Department (PMD) as shown in Figs 14 and 15. The base wind speed is taken as 12 m/s, whereas wind speed varies between 3 and 13 m/s as shown in Fig 14. A maximum wind speed of 12.4 m/s is achieved between 4 and 5 h, and the minimum wind speed of 3.1 m/s is captured during 2–3 and 11–13 h.

The ambient temperature and solar irradiance used for the case study are shown in Fig 15. At nighttime, the low temperature is recorded. During 0–6 h, the temperature continues to decrease, and a minimum temperature of 27°C is obtained during 5–6 h. After 6 h, the temperature continues to increase, and a maximum temperature of 41°C is attained during 14–18 h. After 18 h, the temperature again gradually decreases until 23 h. The irradiance level varies over the 24 h cycle depending upon the appearance of the sun. In the absence of the sun—i.e., during 0–5.5 h and 19–24 h—the irradiance level is zero. After 5.5 h, the irradiance level increases, and a maximum irradiance level of 1058 W/m^2^ is obtained during 11–13 h.

**Fig 16:**
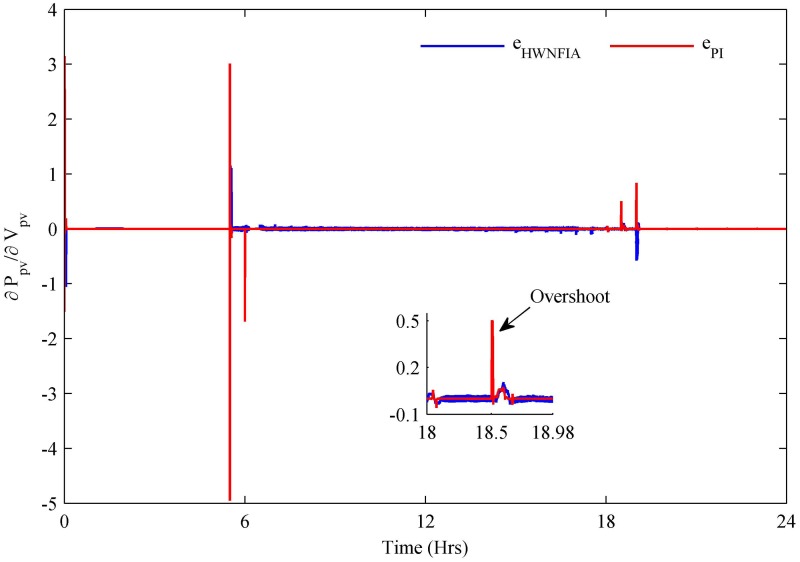
PV MPPT error.

The HWNFIAC for the PV system tracks the MPP by keeping the slope close to zero. To analyze the performance of the HWNFIAC, a PI controller is also used to track the MPP of the PV system. It is clear from Fig 16 that under rapid change of atmospheric conditions, the HWNFIAC achieves the MPP quickly. The PI controller also tracks the PV MPP, but when the sudden change in atmospheric conditions occurs, the PI controller loses its control for an instant of time, which results in spikes—e.g., at 0, 5.5, 6, 18.5 and 19 h. The accuracy and stability of the PV HWNFIAC is better than the PI controller.

**Fig 17:**
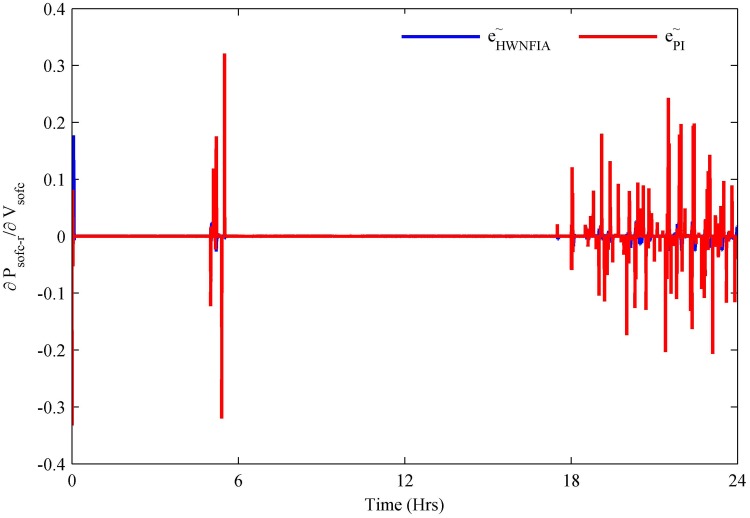
SOFC hydrogen utilization error.

The HWNFIAC for the SOFC is used to obtain a swift response by controlling the molar flow of input hydrogen. The power drawn from the SOFC is proportional to the molar flow of hydrogen. In the case of load variations, after a short transient period, the HWNFIAC quickly achieves the stable condition compared to the PI controller as shown in Fig 17. The PI controller takes time and fluctuates more for load variations. The HWNFIAC provides a better control than PI.

**Fig 18:**
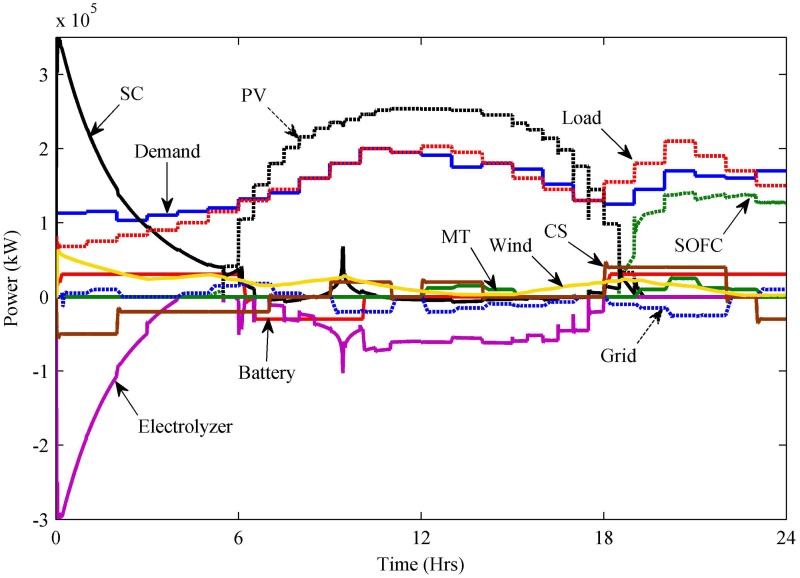
Generated and consumed power.

The generated and consumed power of the stated HPS is shown in Fig 18. The power generated from renewable energy sources—i.e., wind and PV—is initially used to satisfy the load. In the absence of renewable power, other sources of HPS participate accordingly to satisfy the load. During nighttime, i.e.—0–2.19 h—the load varies between 54 and 83 kW. For this time interval, the wind power fluctuates between 31 and 63 kW, and the PV power is completely unavailable. Initially, the battery and SC are considered fully charged, so these two backup sources deliver the power to the load. The grid delivers the rest of the power up to 10 kW to the load. The load is met by wind, battery, SC and grid power, so there is no need to take power from the SOFC and MT. Any excess power in the system is utilized by the electrolyzer to keep the system stable. For the time interval 2.1–5.5 h, the load increases to 83–115 kW. During this time interval, the wind power decreases to 31–24 kW for the interval 2.1–3 h and then increases to 24–29.7 kW for the interval 3–5 h. Between 5 and 5.5 h, the wind power again decreases to 29.7–26.8 kW. PV power is still inaccessible for this interval. The battery and SC remain in discharge mode during this time interval. The grid delivers a maximum of 15 kW between 4 and 5.5 h, and the SOFC delivers a maximum of 7.3 kW between 5 and 5.5 h. The MT is kept off during this time interval and the electrolyzer utilizes the surplus power from the system.

During the time interval 5.5–10 h, the load increases to 115–200 kW. For this time interval, the wind power varies, a minimum wind power of 15 kW is captured at t = 6.7 h, and a maximum wind power of 28 kW is acquired at t = 9.5 h. PV power is now available, and a maximum of 246 kW is obtained. The battery is in discharge mode until 6.2 h and then remains in charge mode for 6.2–10 h. The SC is in discharge mode for 5.5–6.5 h, but between 6.5 and 7 h, the SC neither utilizes nor delivers the power. Between 7 and 8.5 h, the SC is in charge mode and then remains in discharge mode for 8.5–10 h. The grid delivers a maximum of 18 kW between 5.5 and 8 h, but the grid takes a maximum of 20 kW during 9–10 h. During this time interval, the SOFC and MT are kept off, because the load is satisfied. The electrolyzer takes all excess power of the system.

During 10–19 h, fluctuating wind gives a maximum of 23 kW at t = 18.79 h. The PV system delivers its maximum power of 253 kW between 11 and 13 h, and then PV power starts decreasing and becomes zero again at t = 19 h. After t = 18 h, the battery delivers the power. The SC remains in charge mode between 10.5 and 17.5 h and then starts discharging after 17.5 h. The SOFC starts delivering the power after t = 18.5 h. During this time interval, the grid takes a maximum of 20 kW. Between 12 and 15.25 h, the MT delivers approximately 15 kW of power. The electrolyzer utilizes excess power. For 19–24 h, the wind power decreases to 21–2.5 kW. PV power is again unavailable. The battery remains in discharge mode and delivers 30 kW of power. The SC delivers the power until t = 19.3 h only. The SOFC delivers maximum power of 140 kW. The MT delivers a maximum of 25 kW between 19 and 23.25 h. The grid takes a maximum of 25 kW power until 22.25 h and then delivers 10 kW of power during 23.25–24 h. The electrolyzer utilizes excess power.

**Fig 19:**
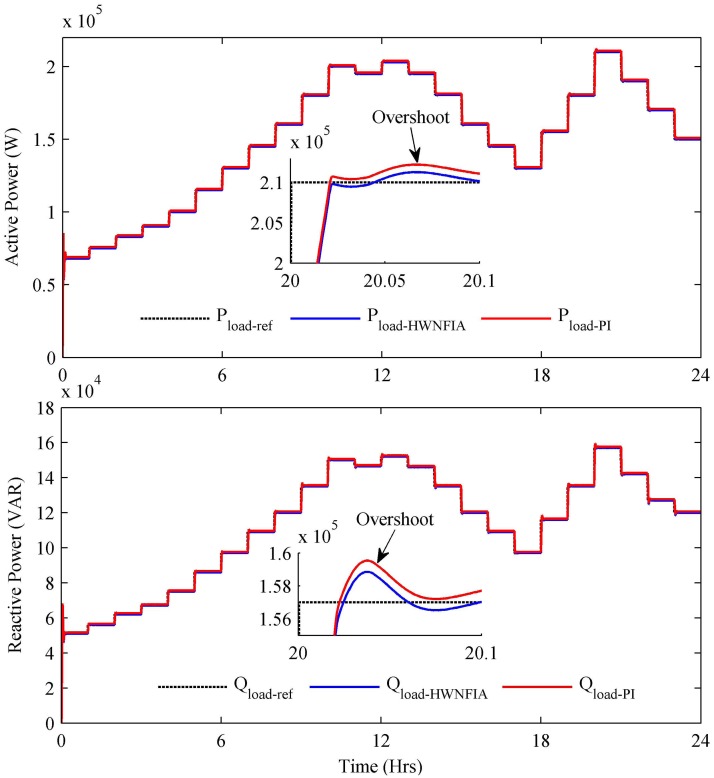
Load active and reactive power.

The reference power shown in Fig 19 is the load required power. At each hour, the load required power is essentially satisfied by the power extracted from the generating sources used in the HPS. The zoomed Figs of active and reactive load show that although the PI controller reduces the steady state error, it increases the overshoot, undershoot and settling time compared to the HWNFIAC as mentioned in Table 8.

**Table 8:** Comparative analysis of controllers.

Photovoltaic	Max. Power (kW)	Overshoot	Undershoot	Steady State Error (kW)	Settling Time (s)
**PV** with No Control	234	0%	-6%	20	0.29
**PV** with PI	242	6%	-18%	12	0.03
**PV** with HWNFIAC	253.6	0%	-2%	0.4	0.01
**SOFC with** PI	140.4	0%	-12%	3	0.31
**SOFC with** HWNFIAC	141.9	0%	-7%	1.5	0.13
**Load (kW)** with PI	210.9	3%	-22%	-1	0.19
**Load (kW)** with HWNFIAC	210	1%	-18%	0	0.1
**Load (kVAR)** with PI	158	2%	-8%	-700	0.139
**Load (kVAR)** with HNFIAC	157	1%	-4%	0	0.119
**Max. Deviation**	**V**_**rms**_	**Frequency**	**V**_**THDs**_	**I**_**THDs**_	
PI	1.53%	0.1748%	4.084%	3.79%	
HWNFIAC	1.32%	0.1443%	3.85%	3.89%	

**Fig 20:**
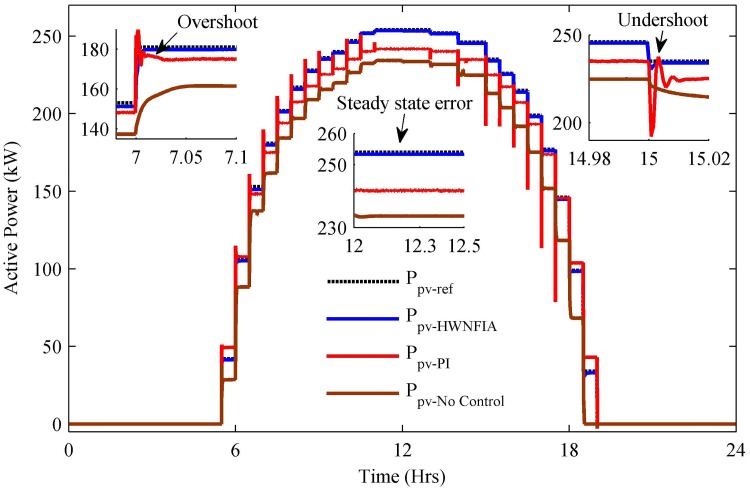
PV output power.

The PV generated power is shown in Fig 20. The PV power is unavailable for 0–5.5 h and 19–24 h—i.e., at nighttime. After t = 5.5 h, the PV power continues to increase until t = 13 h, where maximum power of 254 kW is captured and then starts decreasing. Fig 20 shows the PV power captured with the HWNFIAC, PI and without any control. The HWNFIAC tracks the reference power with minimum overshoot, undershoot, steady state error and settling time compared to PI and without control as given in Table 8. At t = 12 h, the peak reference PV power is 254 kW, whereas the HWNFIAC acquires 253.6 kW; PI achieves 242 kW and without control 234 kW. According to Table 8, although the overshoot and undershoot in the case without control are less than with the PI control, the steady-state error is much higher than with PI control. In the zoomed sub-figures depicted in Fig 20, the overshoot, undershoot and steady-state error with the HWNFIAC, with the PI and without control are clearly shown.

**Fig 21:**
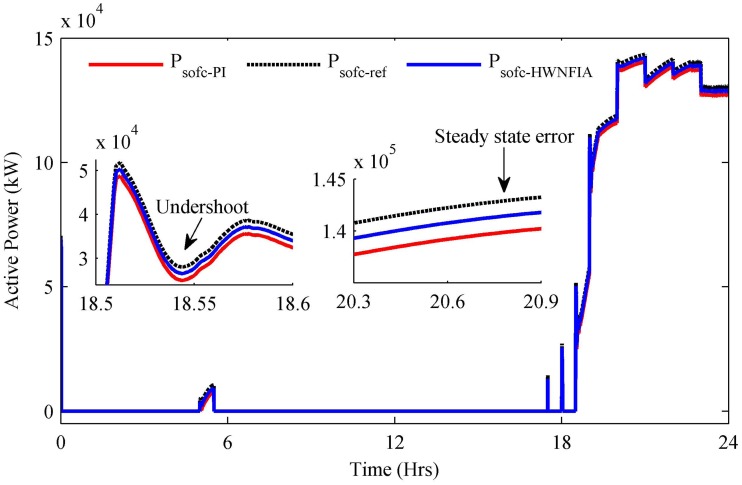
SOFC output power.

The reference power is the power required by the SOFC as shown in Fig 21. The reference power is tracked by both controllers—i.e., HWNFIAC and PI. Fig 21 shows that the HWNFIAC tracks the reference power more quickly compared to the PI controller. The undershoot, steady-state error and settling time of the HWNFIAC are less than with the PI controller as mentioned in Table 8. The zoomed Figs of undershoot and steady-state error are also presented in Fig 21.

**Fig 22:**
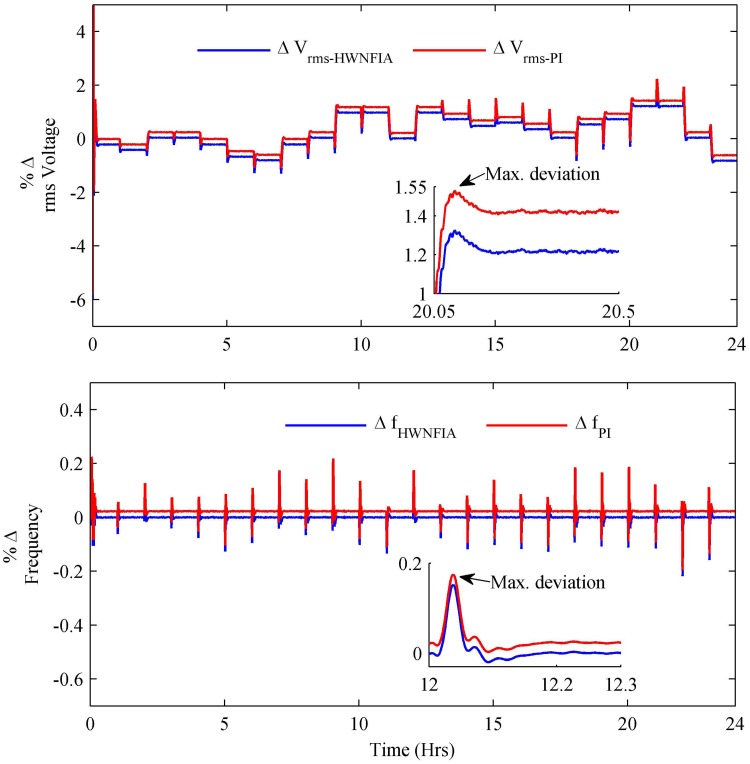
Percentage change in load voltage and frequency.

In the HPS, power quality is the key issue to be addressed. Power quality evaluates the fitness of electric power to consumer devices and is measured in terms of voltage regulation, frequency stabilization and total harmonic distortion. To ensure power quality, assumptions are applied according to the IEEE 1547 standard [43]. In Fig 22, the percentage change in load RMS voltage and fundamental frequency are shown. The percentage change in both the load RMS voltage and fundamental frequency are in their acceptable limits for both HWNFIA and PI controllers, which ensures the system stability and quality power. The maximum deviation of load RMS voltage with the HWNFIAC and PI are 1.32% and 1.53%, respectively. Similarly, the maximum deviation of the load frequency with the HWNFIAC and PI are 0.1443% and 0.1748%, respectively, as mentioned in Table 8.

**Fig 23:**
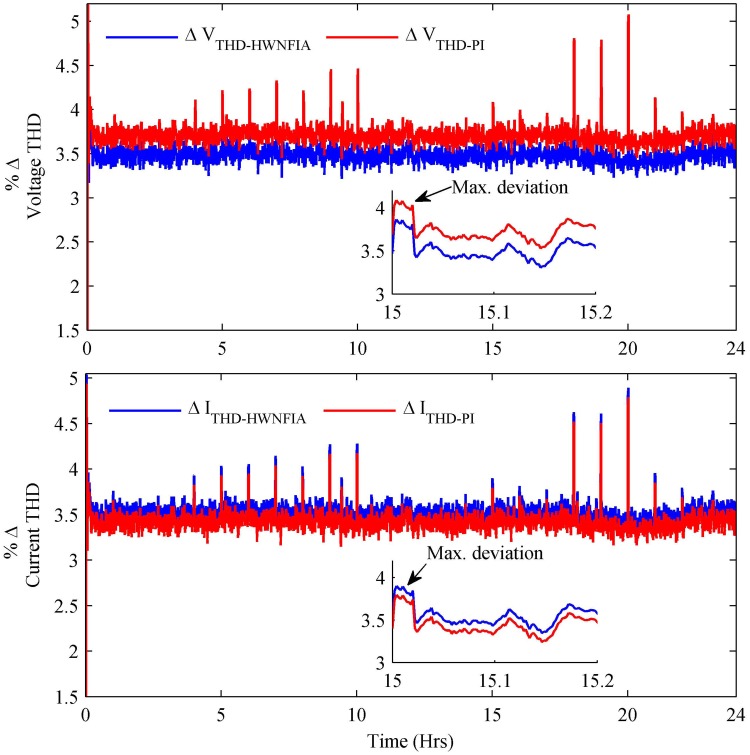
Percentage change in voltage THD and current THD.

In Fig 23, the THD for voltage and current are shown, which are in their standard limits for both HWNFIAC and PI controllers. The maximum deviation of voltage THD with HWNFIAC and PI are 3.85% and 4.084%, respectively. Similarly, the maximum deviation of current THD with HWNFIAC and PI are 3.89% and 3.79%, respectively. It is clear from Table 8 that the maximum deviation of RMS voltage, fundamental frequency and voltage THD are less in the case of the HWNFIAC compared to the PI controller.

## 5. Conclusions

This paper presents a grid-connected HPS consisting of wind turbine, PV, SOFC, electrolyzer, battery storage system, SC and MT generating sources to satisfy a dynamic residential load. The dynamic models of all components of the HPS are discussed. In the HPS, the continuous and sustainable power supply to the load is achieved by supervisory control. The results prove that power is well managed in the HPS under rapid change of atmospheric and dynamic load conditions.

The main contribution of this research work is to extract the maximum power from the PV system and obtain a swift response of the SOFC. Two Hermite wavelet embedded NeuroFuzzy indirect adaptive control systems are implemented to track the MPPT of the PV and swift response of the SOFC. The HWNFIAC for the PV system quickly tracks the MPP, and the HWNFIAC for the SOFC accurately achieves the swift response of the SOFC. To track the MPPT of the PV system and obtain the swift response of the SOFC, Hermite wavelet embedded NeuroFuzzy indirect adaptive control systems have higher precision than conventional PI control systems in terms of overshoot, undershoot, steady-state error and settling time. Hermite wavelet embedded NeuroFuzzy indirect adaptive control systems for both PV and SOFC have smaller overshoot, undershoot, steady-state error and settling time compared to conventional PI controllers.

## Future work

The future work is to implement the Chebyshev wavelet-based NeuroFuzzy indirect adaptive control system to track the MPP of the wind generation system.

## References

[1] Conti J, Holtberg P, Doman LE, Smith KA, Sullivan JO, Vincent KR, et al. International energy outlook 2011. US Energy Information Administration, Technical Report No. DOE/EIA-0484. 2011 Sep.

[2] Mohammed YS, Mustafa MW, Bashir N. Hybrid renewable energy systems for off-grid electric power: Review of substantial issues. Renewable and Sustainable Energy Reviews. 2014 7 31;35:527–39.

[3] Wu X, Hu X, Moura S, Yin X, Pickert V. Stochastic control of smart home energy management with plug-in electric vehicle battery energy storage and photovoltaic array. Journal of Power Sources. 2016 11 30;333:203–12.

[4] Nelson DB, Nehrir MH, Wang C. Unit sizing and cost analysis of stand-alone hybrid wind/PV/fuel cell power generation systems. Renewable energy. 2006 8 31;31(10):1641–56.

[5] Wang C, Nehrir MH. Power management of a stand-alone wind/photovoltaic/fuel cell energy system. IEEE transactions on energy conversion. 2008 9;23(3):957–67.

[6] Uzunoglu M, Onar OC, Alam MS. Modeling, control and simulation of a PV/FC/UC based hybrid power generation system for stand-alone applications. Renewable Energy. 2009 3 31;34 (3):509–20.

[7] Hosseini M, Dincer I, Rosen MA. Hybrid solar—fuel cell combined heat and power systems for residential applications: energy and exergy analyses. Journal of Power Sources. 2013 1 1;221:372–80.

[8] Ahmed J, Salam Z. An improved perturb and observe (P&O) maximum power point tracking (MPPT) algorithm for higher efficiency. Applied Energy. 2015 7 15;150:97–108.

[9] Sivakumar P, Kader AA, Kaliavaradhan Y, Arutchelvi M. Analysis and enhancement of PV efficiency with incremental conductance MPPT technique under non-linear loading conditions. Renewable Energy. 2015 9 30;81:543–50.

[10] Gokmen N, Karatepe E, Ugranli F, Silvestre S. Voltage band based global MPPT controller for photovoltaic systems. Solar Energy. 2013 12 31;98:322–34.

[11] Mutoh N, Inoue T. A control method to charge series-connected ultraelectric double-layer capacitors suitable for photovoltaic generation systems combining MPPT control method. IEEE Transactions on Industrial Electronics. 2007 2;54(1):374–83.

[12] Wu XJ, Zhu XJ, Cao GY, Tu HY. Predictive control of SOFC based on a GA-RBF neural network model. Journal of Power Sources. 2008 4 15;179(1):232–9.

[13] Grujicic M, Chittajallu KM, Law EH, Pukrushpan JT. Model-based control strategies in the dynamic interaction of air supply and fuel cell. Proceedings of the Institution of Mechanical Engineers, Part A: Journal of Power and Energy. 2004 11 1;218 (7):487–99.

[14] Stiller C, Thorud B, Bolland O, Kandepu R, Imsland L. Control strategy for a solid oxide fuel cell and gas turbine hybrid system. Journal of power sources. 2006 7 14;158 (1):303–15.

[15] Pukrushpan JT, Stefanopoulou AG, Peng H. Modeling and control for PEM fuel cell stack system. InProceedings of the 2002 American Control Conference (IEEE Cat. No. CH37301) 2002 May 8 (Vol. 4, pp. 3117–3122). IEEE.

[16] Aguiar P, Adjiman CS, Brandon NP. Anode-supported intermediate-temperature direct internal reforming solid oxide fuel cell: II. Model-based dynamic performance and control. Journal of Power Sources. 2005 9 9; 147 (1):136–47.

[17] Oh SR, Sun J, Dobbs H, King J. Model predictive control for power and thermal management of an integrated solid oxide fuel cell and turbocharger system. IEEE Transactions on Control Systems Technology. 2014 5; 22 (3):911–20.

[18] Gong W, Cai Z. Parameter optimization of PEMFC model with improved multi-strategy adaptive differential evolution. Engineering Applications of Artificial Intelligence. 2014 1 31;27:28–40.

[19] Harrag A, Messalti S. Variable step size modified P&O MPPT algorithm using GA-based hybrid offline/online PID controller. Renewable and Sustainable Energy Reviews. 2015 9 30;49:1247–60.

[20] Ou TC, Hong CM. Dynamic operation and control of microgrid hybrid power systems. Energy. 2014 3 1;66:314–23.

[21] Khan L, Lo KL, Jovanovic S. Hybrid GA neuro-fuzzy damping control system for UPFC. COMPEL-The international journal for computation and mathematics in electrical and electronic engineering. 2006 10 1;25 (4):841–61.

[22] Rezk H, Eltamaly AM. A comprehensive comparison of different MPPT techniques for photovoltaic systems. Solar Energy. 2015 2 28; 112:1–1.

[23] Badar R, Khan L. Coordinated Adaptive Control of Multiple Flexible AC Transmission Systems using Multiple-input—Multiple-output Neuro-fuzzy Damping Control Paradigms. Electric Power Components and Systems. 2014 6 11;42 (8):818–30.

[24] Badar R, Khan L. Neurofuzzy Based Fully Adaptive Indirect Controls for SSSC: A Comparative Analysis. InProceedings of the 2013 11th International Conference on Frontiers of Information Technology 2013 Dec 16 (pp. 95–100). IEEE Computer Society.

[25] Badar R, Khan L. Hybrid neuro-fuzzy legendre-based adaptive control algorithm for static synchronous series compensator. Electric Power Components and Systems. 2013 7 4;41 (9):845–67.

[26] Khan L, Badar R. Hybrid adaptive neuro-fuzzy B-spline—based SSSC damping control paradigm using online system identification. Turkish Journal of Electrical Engineering & Computer Sciences. 2015 3 16;23(2):395–420.

[27] Tao G. Adaptive control design and analysis. John Wiley & Sons; 2003 7 9.

[28] Sastry S, Bodson M. Adaptive control: stability, convergence and robustness. Courier Corporation; 2011.

[29] Mellit A, Kalogirou SA. ANFIS-based modelling for photovoltaic power supply system: A case study. Renewable Energy. 2011 1 31;36 (1):250–8.

[30] Entchev E, Yang L. Application of adaptive neuro-fuzzy inference system techniques and artificial neural networks to predict solid oxide fuel cell performance in residential micro-generation installation. Journal of Power Sources. 2007 6 30;170 (1):122–9.

[31] Abu-Rub H, Iqbal A, Ahmed SM, Peng FZ, Li Y, Baoming G. Quasi-Z-source inverter-based photovoltaic generation system with maximum power tracking control using ANFIS. IEEE Transactions on Sustainable Energy. 2013 1;4(1):11–20.

[32] Wu XJ, Zhu XJ, Cao GY, Tu HY. Nonlinear modeling of a SOFC stack based on ANFIS identification. Simulation Modelling Practice and Theory. 2008 4 30;16(4):399–409.

[33] Wang R, Qi L, Xie X, Ding Q, Li C, Ma CM. Modeling of a 5-cell direct methanol fuel cell using adaptive-network-based fuzzy inference systems. Journal of Power Sources. 2008 12 1;185(2):1201–8.

[34] Tarek B, Said D, Benbouzid ME. Maximum power point tracking control for photovoltaic system using adaptive neuro-fuzzy “ANFIS”. InEcological Vehicles and Renewable Energies (EVER), 2013 8th International Conference and Exhibition on 2013 Mar 27 (pp. 1–7). IEEE.

[35] Haque ME, Negnevitsky M, Muttaqi KM. A novel control strategy for a variable-speed wind turbine with a permanent-magnet synchronous generator. IEEE Transactions on Industry Applications. 2010 1;46(1):331–9.

[36] Zheng L, Zhang L, Zhu J, Wang G, Jiang J. Co-estimation of state-of-charge, capacity and resistance for lithium-ion batteries based on a high-fidelity electrochemical model. Applied Energy. 2016 10 15;180:424–34.

[37] Zhang L, Hu X, Wang Z, Sun F, Dorrell DG. Experimental impedance investigation of an ultracapacitor at different conditions for electric vehicle applications. Journal of Power Sources. 2015 8 1;287:129–38.

[38] Zhang L, Hu X, Wang Z, Sun F, Dorrell DG. Fractional-order modeling and State-of-Charge estimation for ultracapacitors. Journal of Power Sources. 2016 5 15;314:28–34.

[39] Lei Z, Zhenpo W, Xiaosong H, Dorrell DG. Residual capacity estimation for ultracapacitors in electric vehicles using artificial neural network. IFAC Proceedings Volumes. 2014 12 31;47(3):3899–904.

[40] Zhang L, Dorrell DG. Genetic Algorithm based optimal component sizing for an electric vehicle. In Industrial Electronics Society, IECON 2013-39th Annual Conference of the IEEE 2013 Nov 10 (pp. 7331–7336). IEEE.

[41] Gupta AK, Ray SS. An investigation with Hermite Wavelets for accurate solution of fractional Jaulent—Miodek equation associated with energy-dependent Schrödinger potential. Applied Mathematics and Computation. 2015 11 1;270:458–71.

[42] Ray SS, Gupta AK. A numerical investigation of time-fractional modified Fornberg—Whitham equation for analyzing the behavior of water waves. Applied Mathematics and Computation. 2015 9 1;266:135–48.

[43] IEEE standard for interconnecting distributed resources with electric power systems. IEEE Std 1547–2003; 1–28.

